# Advances and challenges in the search for new treatments for Chagas disease

**DOI:** 10.1590/0074-02760250299

**Published:** 2026-07-10

**Authors:** Ana Maria Murta Santi, Davi Alvarenga Lima, Silvane Maria Fonseca Murta

**Affiliations:** 1 Fundação Oswaldo Cruz-Fiocruz, Instituto Carlos Chagas, Curitiba, PR, Brasil; 2 Fundação Oswaldo Cruz-Fiocruz, Instituto René Rachou, Belo Horizonte, MG, Brasil

**Keywords:** Chagas disease, Trypanosoma cruzi, treatment, molecular targets

## Abstract

The treatment of Chagas disease (CD) has relied for more than five decades on two drugs, benznidazole (BZ) and nifurtimox (NTX), both with significant limitations and severe adverse effects. Their limited efficacy during the chronic phase underscores the urgent need for new chemotherapeutic strategies. The pursuit of new therapeutic agents for CD is focused on identifying molecular targets essential for *Trypanosoma cruzi* survival that are either absent or highly divergent in human, thereby enhancing selectivity and minimising off-target toxicity. These targets exploit the parasite's unique biology at multiple levels. Key metabolic vulnerabilities include: ergosterol biosynthesis, a sterol pathway distinct from human cholesterol metabolism; glycosomal metabolism, reflecting parasite´s unique compartmentalisation of glycolysis; and redox homeostasis, which depends on trypanothione rather than glutathione. Additional promising avenues involve the parasite's genetic and epigenetic regulation, mRNA processing and translational control. Furthermore, virulence-associated factors, and specific enzymes such as type I nitroreductase (NTR-1) can be exploited for selective prodrug activation. The complex genomic organisation and pronounced plasticity of *T. cruzi* complicate the identification of novel therapeutic targets. The abundance of proteins annotated as hypothetical or of unknown function further obscure critical metabolic pathways that could serve as druggable targets. In this context, the discovery of new drugs for CD strategically integrates phenotypic, target-based, and computational approaches, all of which require rigorous validation through preclinical *in vitro* and *in vivo* studies. Although modern approaches have yielded several promising lead compounds, successfully controlling CD will also depend on overcoming socioeconomic and access-related barriers to ensure that new therapies reach the populations most affected by this neglected tropical disease.

Chagas disease (CD) is caused by the protozoan parasite *Trypanosoma cruzi*, which can be transmitted by triatomine bugs, through contact with their faeces containing the infective metacyclic trypomastigotes. In addition to this vector-borne route, transmission can occur congenitally, via blood transfusion, organ transplantation, laboratory accidents, or through ingestion of food or beverages contaminated with the parasite. It is estimated to affect 7 million people worldwide, primarily poor populations in Latin America.^1^

Fighting CD faces several critical challenges, including: difficulties in controlling insect vectors, despite notable progress in recent years; increasing rates of oral transmission; low awareness of the disease among both the general population and healthcare professionals; the absence of a vaccine; and inadequate screening programs that contribute to frequent late diagnosis. There is also an urgent need for improved point-of-care diagnostic tools, as well as more effective and less toxic therapeutic options. The emergence of drug-resistant *T. cruzi* strains; high medical costs, and recurrent drug shortages further complicate disease management. Moreover, limited healthcare infrastructure outside urban centres; the fact that few patients are diagnosed in time and even fewer receive adequate treatment; difficulties in rehabilitating Chagas-related cardiomyopathy; and the lack of reliable prognostic biomarkers remain major obstacles to effective control and patient care.^2^ ^3^ ^4^ ^5^ ^6^ ^7^

CD is a potentially life-threatening disease with debilitating chronic symptoms that can affect the cardiovascular, digestive, and central nervous systems.^5^ Following infection, patients develop an acute phase that may be symptomatic or asymptomatic. Once this phase subsides, the disease progresses to the chronic phase. In 70% of cases, this remains an indeterminate (asymptomatic) form that can last a lifetime, while 30% of patients develop clinical disease, manifesting as cardiac, gastrointestinal, or combined symptoms.^5^ Immunosuppression can cause reactivation of CD, with symptoms resembling the acute phase or presenting as neurological manifestations.^5^ Over time, individuals with cardiac involvement frequently experience progressive morbidity and mortality, which may be exacerbated by comorbid age-related cardiovascular risk factors.^8^ CD carries significant psychosocial consequences—patients often face stigma, diminished health-related quality of life, and substantial psychological impact.^9^

Benznidazole (BZ) and nifurtimox (NTX) are currently the only two drugs available for the treatment of CD, but both exhibit suboptimal efficacy and considerable toxicity.^1^ Treatment during the acute phase achieves high rates of parasitological cure and seroconversion, while its effectiveness decreases substantially in the chronic phase. Children show better therapeutic responses, with greater tolerability and improved outcomes compared to adults.^10^ Despite these limitations, several studies have demonstrated that BZ therapy in chronic CD provides meaningful clinical benefits, particularly in the earlier stages of the disease, by reducing parasite burden, delaying clinical progression, and lowering the risk of cardiovascular complications and mortality.^11^ ^12^ ^13^ ^14^ Moreover, BZ administration in the chronic phase has been associated with improvements in immune responses and myocardial function.^15^

The significant toxicity of the available drugs contributes to high treatment discontinuation rates, particularly among adult patients.^16^ Moreover, parasite elimination does not reverse established cardiovascular or digestive damage, underscoring the need for complementary strategies to manage disease progression.^17^

Here, we propose to explore recent research efforts to discover new treatment strategies for CD, ranging from new BZ formulations and combination therapies to the pursuit of novel molecular targets and drugs in preclinical and early clinical development at DNDi.

## Methods used for drug discovery against CD

The discovery of new drugs for CD integrates phenotypic, target-based, and computational approaches, combined with preclinical *in vitro* and *in vivo* validation.

*Phenotypic (cell-based) screening* - Phenotypic screening is regarded as the most reliable strategy for identifying new anti-*T. cruzi* compounds, since it evaluates drug activity directly against intracellular amastigotes in mammalian cells, capturing host–parasite interactions, permeability, and metabolic activation.^18^ ^19^ ^20^ Early assays used colorimetric, fluorimetric and bioluminescence readouts,^21^ while more recent high-throughput screening (HTS) platforms with automated microscopy and reporter parasites now enable rapid testing of thousands of molecules.^22^ ^23^ ^24^ High-content imaging provides single-cell resolution of parasite load and host cell viability, while live-cell imaging and time-to-kill assays distinguish between trypanocidal and trypanostatic effects.^25^ ^26^ ^27^ ^28^ ^29^

*Target-based drug discovery* - Target-based approaches have gained importance in CD research due to advances in *T. cruzi* genomics, proteomics, and genome-editing tools, which have facilitated the validation of essential parasite proteins as druggable targets. Among the most extensively studied are cruzain, the major cysteine protease required for parasite survival and host invasion; sterol 14α-demethylase (CYP51), a key enzyme in ergosterol biosynthesis; trypanothione reductase (TR), central to parasite redox balance; and more recently, the proteasome, cytochrome b, and cleavage and polyadenylation specificity factor 3 (CPSF3).^30^ Structure-guided drug design and computational chemistry have enabled the identification of highly potent inhibitors for these targets, and several compounds have advanced to preclinical or clinical evaluation. Nevertheless, translation has been challenging, as demonstrated by the clinical failure of azole antifungals such as posaconazole and ravuconazole, which showed strong *in vitro* and *in vivo* efficacy against CYP51 but failed to outperform BZ in patients.^26^ ^31^ This discrepancy highlights the need for a critical re-evaluation of preclinical models and protocols.^31^ For a detailed discussion, see sections “*In vivo* studies”, and “Inhibiting the ergosterol biosynthesis pathway”.

*Drug repositioning* - Drug repositioning represents a pragmatic strategy for neglected diseases such as CD, where limited financial incentives constrain traditional drug development. By leveraging existing compounds with established pharmacokinetics and safety profiles, repositioning can shorten timelines and reduce costs compared to *de novo* discovery. Structure-based screening has been particularly valuable, FDA-approved compounds such as ciprofloxacin, naproxen, and folic acid presented anti-*T. cruzi* activity.^32^ Repositioning is attractive when pursued in combination with phenotypic assays and rational selection, offering a rapid entry point for candidate prioritisation and providing scaffolds for medicinal chemistry optimisation. Several repositioned antifungals were tested against CD, notably posaconazole and ravuconazole (as referenced in section “Target-based drug discovery”).^26^ ^31^

*Computational approaches* - *In silico* methods have become indispensable tools for drug discovery against *T. cruzi*, accelerating the identification, optimisation, and prioritisation of compounds before experimental validation. Computer-aided drug design (CADD) integrates diverse strategies that streamline the drug discovery pipeline and reduce both time and cost.^22^ ^30^ Virtual screening is one of the most widely used approaches and includes ligand-based, structure-based, and systems-based methodologies. Ligand-based screening exploits cheminformatics and quantitative structure-activity relationship (QSAR) models to predict activity from chemical features, while structure-based screening relies on molecular docking to estimate the binding affinity of compounds to validated parasite targets.^33^ ^34^ ^35^ Systems-based approaches, such as proteochemometric modelling (PCM), extend QSAR by incorporating both ligand and protein features, enabling predictions of ligand-target interactions even in the absence of complete structural information.^33^ ^34^ ^35^ Pharmacophore modelling further supports rational design by identifying the essential chemical features required for activity, guiding the modification of lead compounds to enhance potency, selectivity, and pharmacokinetic properties. In parallel, machine learning and artificial intelligence (AI) approaches are increasingly applied to predict biological activity, toxicity, and absorption, distribution, metabolism, excretion, toxicity (ADMET) parameters, thereby allowing, in earlier drug discovery steps, to integrate information from both ligands and targets to predict bioactivity or binding affinity.^26^

*Omics and mode-of-action studies in T. cruzi* - Omics technologies provide crucial, system-wide insights into drug action and resistance in *T. cruzi*. These tools map how compounds perturb parasite biology and identify the adaptive responses that arise under pharmacological pressure. Current evidence positions BZ resistance in *T. cruzi* as a multigenic trait, as demonstrated by several omics studies. Proteomic profiling of resistant strains reveals upregulation of molecular chaperones and proteins involved in redox metabolism, including key antioxidant enzymes.^36^ Metabolomic profiling demonstrated significant remodelling of energy metabolism and lipid utilisation during BZ treatment.^37^ ^38^ Meanwhile, transcriptomic analyses reveal differential expression of genes linked to oxidative stress response and DNA repair in BZ-resistant *T. cruzi* strains.^39^ ^40^ ^41^ ^42^ Collectively, these findings show that resistance in *T. cruzi* is mediated by broad metabolic and transcriptional reprogramming rather than single-gene alterations. This multigenic aspect will be explored in further detail in section “Drug resistance mechanisms”.

The development of CRISPR/Cas9-based genome editing in *T. cruzi* has opened new avenues for validating drug targets and dissecting resistance mechanisms.^43^ ^44^ ^45^ Together with the Omics, these studies offer a foundation for rational drug development and the identification of biomarkers predictive of treatment outcomes.

*In vivo studies* - Promising drug candidates identified through *in vitro* screening are subsequently advanced to *in vivo* models, where their safety and ability to significantly reduce parasitaemia are evaluated. This translational step, however, remains challenging due to the lack of standardised animal models. The use of different parasite strains, host species, and infection routes to address specific aspects of the disease complicates cross-study comparisons and slows the drug discovery process.^20^ Given that no single model fully recapitulates CD, a more realistic and effective strategy is the development of multiple robust and standardised models rather than the pursuit of a single ideal system.^20^

Murine models are the most widely employed. According to the protocol proposed by Romanha et al.,^24^ ^46^ murine drug screening follows a multi-stage approach in which promising compounds are first evaluated in acute infection models to assess efficacy and define optimal dosing. Candidates that show favourable profiles are then tested in chronic infection models, where their ability to prevent parasite relapse following immunosuppression is assessed, thereby mimicking the clinical challenge of eliminating tissue-resident parasites. In light of current knowledge regarding the dynamic and tissue-specific colonisation of *T. cruzi* during chronic infection, the use of chronic models is now strongly recommended for drug evaluation.^24^

Canine and non-human primate models also offer important translational value for human CD. Dogs naturally develop chronic cardiac manifestations similar to those observed in humans, while non-human primates are physiologically the closest model to humans. Nevertheless, their use is constrained by high costs, logistical complexity, and ethical considerations.^20^

Finally, the use of genetically modified parasites expressing luciferase or fluorescent reporters such as tdTomato represents a major advance in drug testing. These systems enable not only high-throughput *in vitro* screening but also non-invasive, longitudinal monitoring of infection dynamics *in vivo*.^21^ ^47^

## Improving the use of BZ

BZ acts as a prodrug that requires activation by trypanosome-specific nitroreductases (NTRs) to exert its trypanocidal effects. This enzymatic reduction produces unstable and highly reactive intermediates, including glyoxal radicals, which damage low-molecular-weight thiols, nucleotides, and macromolecules such as DNA, lipids, and proteins.^38^ ^48^ ^49^ ^50^

Although BZ has been used since the 1970's, many aspects of its pharmacokinetics are not completely understood, and the optimal dosing regimen continues to be debated.^10^ ^51^ ^52^ ^53^ ^54^ Therapeutic efficacy is highest in the acute phase, while age and sex significantly influence drug disposition — children showing better tolerance and response than adults.^10^ ^53^ Ramos et al. summarised trials from 1994-2021, emphasising ongoing efforts to refine BZ dosing.^22^ ^53^ ^55^ ^56^

BZ efficacy can be compromised by the natural resistance of some *T. cruzi* strains,^57^ and by the presence of dormant, non-replicative parasite forms that exhibit intrinsic tolerance to treatment.^58^ These critical issues — drug resistance and parasite dormancy — are further discussed in the section “Challenges in CD treatment”.

To overcome BZ limitations, several innovative strategies are being pursued. One approach focuses on designing novel BZ analogues and derivatives to enhance potency and selectivity. Examples include structural modifications in the phenyl, linker, or benzimidazole regions,^59^ development of metal-based complexes (*e.g.*, silver or copper derivatives),^60^ molecular hybrids combining BZ with other bioactive scaffolds such as eugenol,^61^ and new nitroimidazole derivatives like 2-nitroimidazole-N-acylhydrazone and 2-nitroimidazole-3,5-disubstituted isoxazole compounds.^62^ ^63^

Another promising avenue involves drug combination therapies, aiming to lower BZ doses, reduce toxicity, shorten treatment duration, and minimise resistance by targeting multiple parasite pathways. Combinations under investigation include antifungal agents (itraconazole, ketoconazole, posaconazole, ravuconazole, voriconazole), arylimidamides, ascorbic acid, acetylsalicylic acid, levamisole, clomipramine, fenofibrate, simvastatin, clofazimine, and benidipine, among others.^56^ ^64^ Additional synergistic partners include fexinidazole and its sulfone metabolite,^65^ atorvastatin,^66^ disulfiram,^67^ ^68^ miltefosine,^69^ chloroquine,^70^ proline racemase inhibitors,^71^ 4-nitrobenzoylcoumarin,^72^ pyrrolopyrimidine series,^73^ candimine,^74^ and amiodarone.^75^ Synergistic combinations of BZ chemotherapy with therapeutic vaccines have also shown encouraging results.^76^ ^77^

Finally, nanotechnology-based delivery systems — including lipid nanoparticles, liposomes, polymeric carriers, and dendrimers — have emerged to improve BZ bioavailability, stability, and safety, while potentially modulating immune responses.^78^ ^79^

## New molecular targets

Several metabolic pathways emerge as promising sources of molecular targets due to their essential roles in parasite biology, their uniqueness compared with other eukaryotes, and their demonstrated association with drug susceptibility or resistance phenotypes.^80^ ^81^ The Figure illustrates the subcellular localisation of potential drug targets in *T. cruzi* amastigotes, while Supplementary data (Table) provides a detailed summary of the corresponding metabolic pathways/drug targets, and their known inhibitors.

**Figure:**
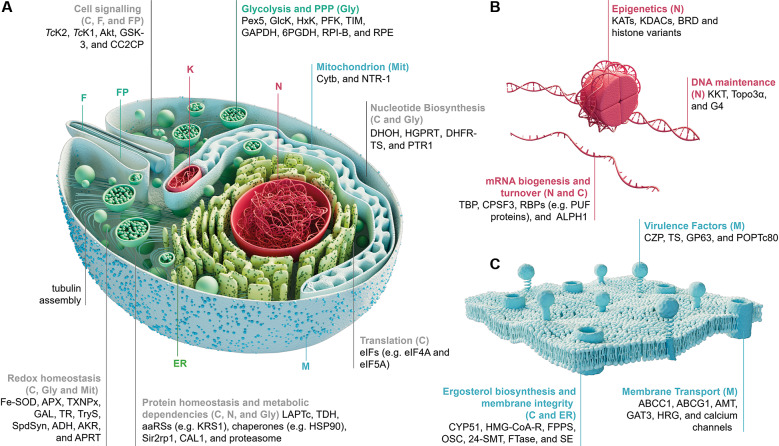
Subcellular localisation of potential drug targets in *Trypanosoma cruzi* amastigotes. (A) Schematic representation (not to scale) of a cross-section of the amastigote stage, showing the primary subcellular localisation of key drug targets. The highlighted pathways include redox and protein homeostasis, translation, cell signalling, and core metabolism within specialised organelles or cytoplasm. Organelles: K: kinetoplast; F: flagellum; FP: flagellar pocket; N: nucleus; ER: endoplasmic reticulum; C: cytosol; M: plasma membrane; Gly: glycosome; Mit: Mitochondrion. (B) Drug targets for genetic information processing. The image (not to scale) highlights targets for DNA replication and maintenance; epigenetic regulation; mRNA biogenesis and turnover. (C) Schematic representation (not to scale) of the cell membrane and associated structures, emphasising targets involved in membrane integrity, membrane transport and virulence factors. aaRSs: Aminoacyl-tRNA synthetases; ABCC1: ATP-binding cassette C1; ABCG1: ATP-binding cassette G1; ADH: alcohol dehydrogenase; AKR: aldo-keto reductase; AKT: Akt-like kinase; ALPH1: ApaH-like phosphatase; AMT: ammonium transporter; APRT: adenine phosphoribosyltransferase; APX: ascorbate peroxidase; BRD: Bromodomains; TcCA: Carbonic anhydrases; CAL1: Adenine phosphoribosyltransferase; CC2CP: cyclic nucleotide-binding/C2-domain protein; CPSF3: cleavage and polyadenylation specificity factor 3; CZP: cruzipain; CYP51: sterol 14α-demethylase; Cytb: Cytochrome b; DHFR-TS: reductase-thymidylate synthase; DHOH: Dihydroorotate dehydrogenase; eIFs: Translation initiation factors; Fe-SOD: Fe-superoxide dismutases; FPPS: Farnesylpyrophosphate synthase; FTase: farnesyltransferase; G4: G-quadruplexes; GAL: galactonolactone oxidase; GAPDH: Glyceraldehyde-3-phosphate dehydrogenase; GAT3: glycosomal ABC transporter; GlcK: glucokinase; GP63: Glycoprotein 63; GSK-3: Glycogen Synthase Kinase-3; HGPRT: hypoxanthine-guanine phosphoribosyltransferase; HMG-CoA-R: hydroxymethylglutaryl-coenzyme A reductase; HRG: Heme Response Gene; HSP90: heat-shock protein 90; HxK: hexokinase; K1: kinase 1; K2: kinase 2; KATs: lysine acetyltransferases; KDACs: lysine deacetylases; KKT: kinetoplastid kinetochore proteins; KRS1: lysyl-tRNA synthetase; LAPTc: acidic M17 leucyl-aminopeptidase; NTR-1: Type I Nitroreductase; OSC: oxidosqualene cyclase; PEX5: Peroxisomal Cargo Receptor; PFK: Phosphofructokinase; POPTc80: serine protease POPTc80 (prolyl oligopeptidase); PTR1: pteridine reductase 1; PUF: Pumilio/FBF proteins; RBPs: RNA Binding Proteins; RPE: ribulose-5-phosphate epimerase; RPI-B Ribose-5-phosphate isomerase B; SE squalene epoxidase; Sir2rp1 cytoplasmic sirtuin; SpdSyn spermidine synthase; TBP: TATA-box binding; TDH: L-threonine dehydrogenase; TIM: triose phosphate isomerase; Topo3α: topoisomerase IIIα; TR: trypanothione reductase; TryS: Trypanothione Synthetase; TS: trans-sialidase; TXNPx: tryparedoxin peroxidases; 24-SMT: Δ24(25)-sterol methyltransferase; 6PGDH: 6-Phosphogluconate dehydrogenase. This illustration was computationally modelled and rendered by Santi, AMM, using Blender 4.0, an open-source software for 3D visualisation.

## Inhibiting the ergosterol biosynthesis pathway

The ergosterol biosynthesis pathway has been extensively explored as drug target in *T. cruzi*. Unlike human cells that produce cholesterol, trypanosomes rely on the synthesis of ergosterol for survival. Disruption of this pathway compromises parasite viability by destabilising membrane structure and interfering with critical signalling processes.^82^

Among the enzymes involved, sterol 14α-demethylase (CYP51), a haem-containing cytochrome P450, plays a central role by catalysing the removal of the 14α-methyl group from sterol precursors to produce functional sterols such as ergosterol.^83^ Inhibition of TcCYP51 exerts a dual lethal effect, depleting ergosterol and causing toxic accumulation of methylated sterol intermediates.^31^ ^83^ Extensive structural and biochemical evidence has validated TcCYP51 as a druggable enzyme, and several azole-based antifungals, including posaconazole, ravuconazole (and its prodrug fosravuconazole), and VNI/VFV derivatives, have shown potent preclinical activity.^84^ ^85^ However, clinical trials in chronic indeterminate disease using posaconazole^86^ and E1224^87^ failed to achieve durable parasitological cure, likely due to their cytostatic — rather than cytocidal — effects, a shortcoming confirmed by both long-term *in vitro* assays and highly sensitive *in vivo* imaging.^88^ ^89^ Furthermore, structural variations in the CYP51 enzyme across *T. cruzi* strains can result in diminished azole binding affinity.^90^ Collectively, these shortcomings led to the deprioritisation of CYP51 as a drug target.

Beyond CYP51, other enzymes in the sterol biosynthesis pathway have also been explored as potential drug targets.^91^ The enzyme hydroxymethylglutaryl-coenzyme A reductase (HMG-CoA-R) is potently inhibited by the statins such as lovastatin and simvastatin. Farnesylpyrophosphate synthase (FPPS) is a key target, inhibited by nitrogen-containing bisphosphonates and Cu(II)-risedronate complexes. Oxidosqualene cyclase (OSC) can be inhibited by aminopropylindene analogues, a phenolic ether of squalene, and *N*-oxides. Δ24(25)-sterol methyltransferase (24-SMT) has been extensively validated as a target using azasterol derivatives as inhibitors. Additionally, the design of benzophenone-based inhibitors has been pursued against farnesyltransferase FTase. Finally, a series of 4-arylthiazolylhydrazones derived from 1-indanones have been identified as potential inhibitors of squalene epoxidase (SE).^91^

## Disrupting membrane transporters

Membrane transporters are essential for *T. cruzi* metabolism and stress adaptation, making them attractive drug targets.^92^ ATP-binding cassette (ABC) transporters are the most studied, mediating xenobiotic efflux and contributing to BZ resistance.^93^ ^94^ *Tc*ABCC1 exports thiols and conjugates, enhancing tolerance to oxidative stress, while *Tc*ABCG1 participates in lipid and sterol transport; both are upregulated in *T. cruzi* resistant strains. Deletion of the glycosomal ABC transporter (GAT3) increased tolerance to hydrogen peroxide but reduced infectivity in mammalian cells, implicating it in oxidative metabolism.^95^

Other membrane transporters also play critical physiological roles in *T. cruzi*. The ammonium transporter (AMT) is essential for parasite growth, differentiation, and osmoregulation.^96^ Calcium channels represent additional targets, as their inhibition by Gaba, Amlod, Pinav, or amiodarone derivatives disrupts parasite signalling and proliferation.^97^ ^98^ Because *T. cruzi* cannot synthesise heme *de novo*, it relies on Heme Response Gene (HRG) transporters for uptake, a process now inhibited by several repurposed compounds.^99^ ^100^

Together, these findings establish membrane transporters as crucial determinants of *T. cruzi* survival and promising targets for new chemotherapeutic strategies.

## Disrupting glycosomal metabolism

Energy metabolism in *T. cruzi* depends mainly on glycolysis and the pentose phosphate pathway (PPP), both compartmentalised within glycosomes, specialised peroxisome-like organelles absent in mammalian cells. This compartmentalisation makes glycosomes unique and attractive drug targets.^101^

Therapeutic strategies include disrupting protein import into the organelle via the peroxin system. The peroxisomal cargo receptor (Pex5) plays a key role in this process and exhibits structural features that may allow the design of selective inhibitors.^102^ Alternatively, key glycosomal enzymes in glycolysis and the PPP can be directly targeted.^103^

## Glycolytic pathway

In *T. cruzi*, the early glycolytic steps occur in glycosomes and display atypical “aerobic fermentation,” where glucose catabolism produces reduced metabolites even under aerobic conditions.^104^ Glucose phosphorylation by glucokinase (GlcK) and hexokinase (HxK) represents the first committed step, and inhibition of these enzymes induces parasite death. Competitive *Tc*GlcK inhibitors such as 3-nitro-2-phenyl-2H-chromene analogues and CBZ-GlcN have shown promising trypanocidal activity.^105^ ^106^

Phosphofructokinase (PFK) catalyses the rate-limiting step of glycolysis and is validated as a drug target. A high-throughput screen of > 330,000 compounds identified para-amidosulfonamide derivatives with potent activity and selectivity due to a unique allosteric pocket absent in the human enzyme.^107^ ^108^ ^109^

Downstream enzymes also provide opportunities for selective inhibition. Triose phosphate isomerase (TIM) exhibits structural differences at the dimer interface compared to the human enzyme, enabling selective targeting by thiazole, benzothiazole, and benzimidazole derivatives.^110^ Glyceraldehyde-3-phosphate dehydrogenase (GAPDH), essential for ATP generation and redox balance, is inhibited by diverse compounds — including adenosine analogues, ruthenium complexes, and anacardic acid derivatives — exploiting active-site differences from the human enzyme.^111^ ^112^ ^113^

## Pentose phosphate pathway

The PPP provides ribose-5-phosphate for nucleotide synthesis and NADPH for redox homeostasis.^114^ 6-Phosphogluconate dehydrogenase (6PGDH) supports the trypanothione antioxidant system and can be selectively inhibited by sulfoxide and hydroxamic analogues.^115^ Ribose-5-phosphate isomerase B (RPI-B) is another promising, parasite-specific target absent in mammals.^101^ Haloacetamide analogues inhibit both TcRPI-B and ribulose-5-phosphate epimerase (RPE), blocking PPP flux and impairing redox balance.^114^

Collectively, these findings validate glycosomal metabolism as a central vulnerability in *T. cruzi*, with multiple enzymatic nodes suitable for selective therapeutic intervention.

## Targeting cytochrome b (Cytb)

Kinetoplastids are protozoans characterised by a unique structure named the kinetoplast — a dense network of mitochondrial DNA (kDNA) within their single, large mitochondrion. This organelle is central to essential processes like oxidative phosphorylation, which generates ATP via the electron transport chain.^116^ ^117^ Within this system, Cytb, mediates electron transfer from ubiquinol to cytochrome c (Cytc) through two catalytic sites: Qi (ubiquinone reduction) and Qo (ubiquinol oxidation).^118^ *Tc*Cytb was validated as a target using chemical genetics. High-throughput screening identified GNF7686, which inhibited parasite growth. Resistant *T. cruzi* lines carried the L197F mutation at the Qi site, conferring resistance to GNF7686 and to antimycin A (also a Qi inhibitor), but not to Qo site inhibitors. Further optimisation led to quinazolinedione derivatives, particularly compound 18, which reduced parasitaemia *in vitro* and *in vivo*, though its low solubility limited dosing potential.^119^ ^120^

## Interfering with nucleotide biosynthesis

The nucleotide metabolism of *T. cruzi* is uniquely targetable, with key vulnerabilities in three core processes: folate metabolism, pyrimidine *de novo* synthesis, and purine scavenging.

## Folate metabolism

Folate metabolism provides one-carbon units from amino acids such as serine, glycine, and methionine for two vital processes: nucleotide biosynthesis and methylation reactions.^121^ Inhibition of this pathway is a validated therapeutic approach across cancer, malaria, and bacterial infections.^122^

In *T. cruzi*, dihydrofolate reductase-thymidylate synthase (DHFR-TS) is a bifunctional enzyme essential for parasite survival. It forms a symmetric dimer, with each monomer containing fused DHFR and TS domains.^123^ The enzyme's essentiality is supported by the impaired growth of heterozygous mutants and the inability to generate a full knockout.^124^ Structural differences in the folate-binding pocket between trypanosomatid and human DHFRs allow for selective inhibitor design.^121^

However, the efficacy of DHFR inhibitors is limited because pteridine reductase 1 (PTR1) compensates for this inhibition. PTR1 has dual function: it reduces biopterins to produce essential reduced pteridines and serves as a metabolic bypass to compensate for inhibited DHFR activity.^125^ Therefore, dual inhibition of DHFR and PTR1 is considered essential for effective chemotherapeutic targeting.^121^ ^125^

A wide range of compounds have been evaluated as inhibitors of trypanosomatid DHFR, with varying degrees of potency and selectivity.^121^ Classical antifolates such as trimethoprim, pyrimethamine, methotrexate, and cycloguanil exhibit weak activity and poor selectivity in trypanosomatids.^123^ Consequently, alternative scaffolds — identified through structure-based design, repurposing, and screening — have gained attention, including quinazolines, pteridine analogues, diaminopyridines, and other antifolate derivatives.^110^ While several compounds display promising activity, achieving potent and selective inhibition of the TcDHFR-PTR1 system remains a major challenge in *T. cruzi* drug development.^125^

## Purine and pyrimidines

Beyond serving as the building blocks of nucleic acids, purines and pyrimidines act as key precursors for numerous metabolites involved in lipid and carbohydrate metabolism.^126^

In *T. cruzi*, the pyrimidine biosynthesis pathway is fully functional, whereas the salvage route is less robust.^127^ Dihydroorotate dehydrogenase (DHOD), a central enzyme in this pathway, is essential for parasite viability, as knockout mutants fail to survive even with pyrimidine supplementation.^127^ ^128^ *Tc*DHOD differs markedly from its human counterpart — it is a cytosolic dimer using fumarate as electron acceptor, while the human enzyme is mitochondrial and ubiquinone-dependent. These biochemical and structural distinctions offer a strong basis for selective drug design. Indeed, orotate analogues and derivatives have yielded potent and selective *Tc*DHOD inhibitors.^33^ ^129^ ^130^ ^131^ Additional inhibitor classes include thiazolidines, arylideneketones, and flavonoids.^110^

Unlike mammals, *T. cruzi* cannot synthesise purines *de novo* and relies entirely on salvage pathways to obtain them from the host.^82^ This dependency makes purine transport and metabolism attractive drug targets. The parasite expresses several phosphoribosyltransferases (PPRTs), including two isoforms of hypoxanthine-guanine phosphoribosyltransferase (HGPRT), *Tc*A and *Tc*C, which catalyse the conversion of purine bases into nucleotides.^132^ Their essential roles in purine salvage have been exploited pharmacologically: allopurinol, a purine analogue, inhibits parasite growth by acting as an alternative substrate for *Tc*HGPRT.^133^ This dependency on purine salvage can also be exploited using other nucleoside analogues, including allopurinol ribonucleoside,^134^ 9-deazainosine,^134^ formycin B,^135^ 7-(4-chlorophenyl)-aminopurinol ribonucleoside,^136^ tubercidin (7-deazaadenosine),^137^ cordycepin (3′-deoxyadenosine),^137^ tubercidin analogues,^138^ ^139^ ^140^ ^141^ and N6-methyltubercidin.^142^

## Exploiting unique redox homeostasis mechanisms

Trypanosomatids face constant oxidative stress but lack classical antioxidant enzymes such as catalase, selenium-dependent glutathione peroxidase, glutathione reductase, and thioredoxin reductase. Instead, their redox homeostasis depends entirely on trypanothione [T(SH)₂], a unique bis-glutathionyl-spermidine molecule that serves as a universal electron donor for peroxide detoxification and DNA precursor synthesis.^143^ In *T. cruzi*, Fe-superoxide dismutases (Fe-SODs) convert O₂⁻ to H₂O₂, which is then removed by ascorbate peroxidase (APX) or tryparedoxin peroxidases (TXNPxs), all fuelled by reduced trypanothione regenerated by trypanothione reductase (TR). This system, which is absent in vertebrates, represents one of the most distinctive and exploitable biochemical features of trypanosomatids.^45^

## Fe-superoxide dismutases (Fe-SODs)

*T. cruzi* expresses compartment-specific Fe-SOD isoforms, unlike humans, which produce Mn- and Cu/Zn-SODs. Overexpression of mitochondrial TcFe-SOD-A correlates with increased infectivity and BZ resistance.^45^ ^144^ Several inhibitor families have been developed — phthalazines, benzo[g]phthalazines, macrocycles, abietic acid derivatives, dithiocarbamates, and Mannich-base arylamines — with optimised imidazole- and pyrrole-benzo[g]phthalazine analogues showing potent trypanocidal activity and selectivity over human SODs.^145^ ^146^ Despite strong preclinical efficacy, no Fe-SOD inhibitor has yet reached advanced development.

## Ascorbate and peroxide detoxification

APX, a heme enzyme, converts H₂O₂ to water using ascorbate as an electron donor.^147^ Because *T. cruzi* synthesises ascorbate *de novo* via galactonolactone oxidase (GAL) — an enzyme absent in humans — TcGAL constitutes a selective vulnerability. It is inhibited by lycorine, benzodioxole-containing chalcones, and allylpolyalkoxybenzenes.^148^ Ascorbate recycling and peroxide detoxification are further supported by the trypanothione-tryparedoxin-peroxidase (TXNPx) system, which eliminates peroxynitrite and organic hydroperoxides; TXNPx is essential and lacks a human counterpart.^91^

## Trypanothione reductase (TR)

TcTR regenerates reduced trypanothione using NADPH. The enzyme is indispensable — its downregulation decreases infectivity, while overexpression enhances stress tolerance.^149^ Structural differences from human glutathione reductase (GR) allow selective inhibition. Active compound classes include nitrothiophenes, benzo[b]thiophenyl-piperidines (BTCPs), sesquiterpene lactones, polyamines, quinones, metal complexes, and Mannich bases.^110^ ^150^ Virtual screening of phenothiazine derivatives identified moderate inhibitors (Zn_C216, Zn_C687), offering leads for optimization.^151^ However, partial TR activity can sustain parasite survival, demanding highly potent inhibitors with complete enzymatic blockade.

## Trypanothione synthetase and polyamine metabolism

Trypanothione synthetase (TryS) catalyses trypanothione synthesis from glutathione and spermidine and is bifunctional, possessing a reverse hydrolase domain.^152^ Paullone-derived inhibitors such as KuOrb39, KuOrb54, and MOL2008 effectively inhibit TryS in trypanosomes and *Leishmania*.^153^ ^154^ The upstream enzyme spermidine synthase (SpdSyn), which produces spermidine from putrescine and dcSAM, is also essential; several *in silico* and *in vitro* hits have been identified as TcSpdSyn inhibitors.^155^ ^156^

## Other redox-associated drug resistance enzymes

Other redox-linked enzymes, including alcohol dehydrogenase (ADH) and aldo-keto reductase (AKR), contribute to BZ *T. cruzi* resistance. Resistant strains of parasite show reduced TcADH and elevated TcAKR expression, correlating with lower ROS accumulation.^157^ ^158^ Adenine phosphoribosyltransferase (APRT) is downregulated in BZ-resistant *T. cruzi* parasites; conversely, its overexpression increases susceptibility to BZ and H₂O₂, suggesting an indirect role in oxidative stress regulation.^40^

## Interfering with DNA maintenance

Kinetoplastids possess a unique molecular machinery for chromosome segregation, distinct from that of other eukaryotes.^159^ In most organisms, the kinetochore — the protein complex that anchors chromosomes to spindle microtubules during mitosis — is composed of highly conserved proteins such as CENP-A and Ndc80. However, trypanosomatids lack all canonical kinetochore genes and instead assemble their kinetochores from a completely novel set of proteins, termed kinetoplastid kinetochore proteins (KKTs).^159^ These proteins are conserved within the Kinetoplastida lineage, suggesting that this unconventional system evolved in their last common ancestor. The druggability of TcKKT proteins is under preliminary investigation; a kinase-biased high-throughput screen identified a small number of active hits, but these compounds require optimisation to improve potency and pharmacokinetics.^160^

Another emerging nuclear target class involves G-quadruplexes (G4s) — stable, four-stranded DNA or RNA structures formed in guanine-rich regions. Genome-wide analyses revealed a high density of potential G4-forming sequences in *T. cruzi*.^161^ Because G4s regulate essential genetic processes, including DNA replication, transcription, and translation, their stabilisation or disruption may represent a novel therapeutic approach, as shown in cancer and viral diseases.

DNA topoisomerase IIIα (Topo3α) is another essential enzyme in *T. cruzi*, responsible for relieving negative supercoils during DNA replication and repair, thereby maintaining chromosomal stability. Thiosemicarbazones derived from 4-chlorophenylthioacetone have been identified as selective Topo3α inhibitors with *in vitro* trypanocidal activity and *in vivo* efficacy in reducing parasitaemia in mice, without host cytotoxicity.^162^ The thiosemicarbazone scaffold, known for its broad pharmacological versatility, continues to show potential in antiparasitic development. Furthermore, novel anthracycline and cyanotriazole-based compounds have emerged as promising scaffolds for targeting trypanosomatid topoisomerases, expanding this underexplored therapeutic avenue.^91^

## Targeting epigenetics

Gene expression in trypanosomatids is controlled not only by DNA sequence but also by epigenetic (DNA and histone modifications) and epitranscriptomic (RNA modifications) mechanisms that determine when and how genes are expressed.^163^

The histone modifications dynamically modulate chromatin structure and transcription. Lysine/arginine methylation, can either activate or repress transcription depending on the residue and context, while histone phosphorylation generally relaxes chromatin by adding negative charge to serine, threonine, or tyrosine residues.^163^ On the other hand, lysine acetylation, regulated by lysine acetyltransferases (KATs) and deacetylases (KDACs), relaxes chromatin and promotes gene activation. Because of their central role, these enzymes have emerged as promising drug targets against parasitic diseases, including CD.^164^ ^165^ ^166^ In *T. cruzi*, the KDAC inhibitor trichostatin A (TSA) and the KAT inhibitor curcumin showed potent anti-proliferative effects across parasite life stages.^166^ ^167^

Bromodomains (BRDs) act as epigenetic “readers” that recognise acetylated lysines. *T. cruzi* encodes at least seven bromodomain-containing factors (BDFs) with distinct subcellular localisations.^168^ The inability to delete Tcbdf1 and Tcbdf3 highlights their essentiality.^168^ Phylogenetic analyses show that *T. cruzi* BRDs are highly divergent from human homologs, providing opportunities for selective drug design. Several bromodomain inhibitors, including A1B4, iBET151, and 1,3,4-oxadiazoles, have been identified, along with compounds such as SGC-CBP30, bromosporine, and I-BRD9, which interact with *Leishmania* BDF5 and show antiparasitic activity.^169^ A recent screen targeting TcBDF2 identified seven new inhibitors; however, their lack of trypanocidal activity highlights the challenge of translating biochemical inhibition into cellular efficacy.^170^ Other epigenetic reader domains, such as YEATS and methyl-binding domains, remain more difficult to target due to their structural promiscuity.^163^

Kinetoplastid histones also differ markedly from those of conventional eukaryotes. Unique histone variants — H2A.Z, H2B.V, H3.V, and H4.V — define strand switch regions (SSRs), marking transcription initiation and termination sites.^171^ ^172^ H2A.Z and H2B.V are essential for parasite viability in *T. brucei* and *L. major*.^172^ Targeting these variants offers diagnostic and therapeutic opportunities: for example, high-affinity DNA aptamers have been developed against the *Leishmania* H2A histone, representing a potential platform for selective molecular probes and anti-parasitic tools.^173^

## Targeting essential processes in mRNA biogenesis and turnover

The early evolutionary divergence of kinetoplastids produced a unique gene expression system, distinct from that of other eukaryotes. Their genes are arranged into polycistronic transcription units that lack canonical promoter elements, making regulation of individual mRNAs almost entirely post-transcriptional.^174^ ^175^

The essential role of transcription in parasite survival is illustrated by actinomycin D, which inhibits *T. cruzi* proliferation by binding DNA and blocking RNA synthesis.^176^ ^177^ Based on this principle, specific transcriptional regulators have emerged as drug targets. The TATA-box binding protein (TcTBP), a universal transcription factor, was recently shown to bind selective inhibitors identified through molecular docking and dynamics. Among them, DB00890 displayed selective affinity for TcTBP over the human enzyme and moderate trypanocidal activity, providing a promising scaffold for optimisation.^178^

The cleavage and polyadenylation specificity factor 3 (CPSF3) processes pre-mRNAs and adds the poly(A) tail, essential for mRNA stability and translation. In kinetoplastids, CPSF3 is indispensable for gene expression, as confirmed in *T. brucei* knockout studies.^179^ ^180^ The benzoxaborole AN15368, an orally bioavailable prodrug activated by parasite carboxypeptidases, potently inhibits *T. cruzi* via TcCPSF3 inhibition. Mutations such as N232H or gene overexpression confer resistance, confirming target engagement.^181^ Notably, AN15368 achieved complete parasitological cure in non-human primates during both acute and chronic infection phases without detectable toxicity.^181^

RNA Binding Proteins (RBPs) control nearly all post-transcriptional steps — from trans-splicing to mRNA transport, translation, and decay — and are thus central to parasite survival.^182^ Although essentiality has been extensively demonstrated in *T. brucei*, ongoing studies in *T. cruzi* suggest critical roles in cell-cycle control and mRNA stability.^183^ Several RBPs are kinetoplastid-specific, including zinc finger, KH domain, and Pumilio/FBF (PUF) proteins. The latter bind 3’-UTRs to regulate mRNA fate, making them attractive targets for structure-guided drug design.^184^

Kinetoplastids also employ a noncanonical mRNA decapping mechanism. Instead of the typical Dcp2-Dcp1-Edc complex, they use an ApaH-like phosphatase (ALPH1), structurally divergent from other eukaryotic enzymes.^185^ This distinctive mechanism represents another parasite-specific vulnerability for selective therapeutic targeting.

## Targeting translation machinery

The absence of gene-specific transcription regulation in trypanosomatids, a consequence of their polycistronic gene organisation, elevates translational control to the principal mechanism for rapid environmental adaptation. Consequently, the translational apparatus is a point of vulnerability in these parasites.

Translation initiation factors (eIFs) are other group of compelling molecular targets, not only due to their critical role in the parasite's stress response^186^ but also because trypanosomatids possess unique eIF4F-like complexes. As reviewed by Freire et al., these complexes are formed by multiple homologues of eIF4E (cap-binding subunit), eIF4G (scaffolding subunit), and eIF4A (RNA helicase).^187^ These factors vary in size, structure, and properties; consequently, many share low homology with their human counterparts.^187^ It was already shown that natural products such as hippuristanol, rocaglates and pateamine A can target eIF4A.^188^

The initiation factor eIF5A is a multifunctional protein that participates in translation elongation and termination, as well as in apoptosis and RNA decay.^189^ Studies comparing resistant and susceptible strains to BZ revealed lower expression of *Tc*eIF5A in resistant populations relative to controls, while overexpression of this gene in resistant parasites increased their susceptibility to BZ by three- to fivefold. These findings suggest that *Tc*eIF5A may participate in processes required for BZ metabolism and trypanocidal activity.^189^

Taken together, these findings demonstrate that functional characterisation of molecular targets involved in fundamental processes such as protein synthesis may uncover less obvious yet highly relevant candidates for drug discovery in CD.

## Exploiting protein homeostasis and metabolic dependencies

Targeting *T. cruzi*'s dependence on host-derived nutrients offers a strategic therapeutic avenue. The acidic M17 leucyl-aminopeptidase (LAPTc) is essential for liberating amino acids such as leucine from host proteins. A recent high-throughput screen identified selective LAPTc inhibitors, with lead compound four displaying potent activity against intracellular amastigotes, validating this metabolic dependency as a drug target.^190^ ^191^ Once acquired, amino acids also feed energy metabolism; under nutrient stress, L-threonine dehydrogenase (TcTDH) catabolises threonine. Inhibition of this enzyme by TCMDC-143160 disrupts energy production and impairs parasite viability.^192^

Aminoacyl-tRNA synthetases (aaRSs) are another vulnerable node in protein biosynthesis. Quinazoline-based inhibitors targeting lysyl-tRNA synthetase (TcKRS1) block lysyl-tRNA formation by binding to its ATP-binding site, resulting in strong phenotypic activity across *T. cruzi*, *T. brucei*, and *L. donovani*.^193^

Protein folding and post-translational modification processes also present high-value targets. The molecular chaperone Hsp90/Hsp83 is critical for parasite growth and stress response.^91^ Inhibition by geldanamycin confirmed its essentiality for cell division,^194^ while the derivative 17-DMAG showed potent *in vitro* activity but lacked *in vivo* efficacy at non-toxic doses.^195^ Similarly, the cytoplasmic sirtuin Sir2rp1, which regulates protein acetylation and parasite infectivity, can be targeted by BZ-derived inhibitors (BNIPs) such as BNIPSpd, showing strong trypanocidal potential.^196^ ^197^ The calcium-binding chaperone calreticulin (TcCAL1) also influences infectivity—its overexpression perturbs metacyclogenesis but enhances host-cell invasion, suggesting a complex role in virulence regulation.^91^

Among the most advanced targets is the proteasome, a multi-subunit complex that degrades ubiquitinated proteins through chymotrypsin-, trypsin-, and caspase-like activities. Proteasomal function is essential for parasite survival and differentiation, and its inhibition leads to accumulation of ubiquitinated proteins, cell-cycle arrest, and death.^30^ ^198^ Notably, the kinetoplastid proteasome diverges sufficiently from the mammalian enzyme to allow selective inhibition. The azabenzoxazole GNF6702 acts as a potent allosteric inhibitor, while imidazopyridines (*e.g.*, GSK3494245) and pyridazinone analogues — optimised using cryo-EM structures — demonstrate strong *in vitro* and *in vivo* efficacy.^199^ ^200^ Additional hits, including TCMDC-143194, triazolopyrimidines, and the virtually screened LCQFTC11, further confirm the proteasome as a clinically validated and evolutionarily conserved therapeutic target for CD.^30^ ^201^ ^202^

## Targeting specific virulence factors

Proteolytic enzymes play central roles in *T. cruzi* biology, mediating nutrition, invasion, immune evasion, and differentiation, making them some of the most intensively investigated molecular targets for chemotherapy in CD.

## Cruzipain

The cysteine protease cruzipain (CZP) is the best-characterised and most validated protease in *T. cruzi*.^30^ It is expressed in all life-cycle stages and is essential for parasite survival and infectivity. Cruzipain contributes to nutrient acquisition, host-cell invasion, immune modulation, and differentiation.^203^ Multiple isoforms with structural diversity complicate inhibitor design, but extensive efforts have yielded a broad spectrum of potent scaffolds, including thio- and semicarbazones, imidazoles, benzimidazoles, coumarins, oxadiazoles, thiazoles, quinoxalines, triazoles, pyrimidines, hydrazones, benzoyl thioureas, and hydroxymethyl ketones.^110^ ^204^ ^205^ ^206^ ^207^ The vinyl sulfone inhibitor K777 advanced to late preclinical testing but was discontinued due to poor tolerability in non-human primates and dogs.^30^ ^208^ Despite this setback, cruzipain remains one of the most promising and pharmacologically validated drug targets in *T. cruzi*.

## Trans-sialidase

Another hallmark virulence factor is the trans-sialidase (TS), a unique enzyme that transfers sialic acid from host glycoconjugates to the parasite surface, forming a protective sialylated coat critical for infectivity.^209^ ^210^ *T. cruzi* lacks *de novo* sialic acid synthesis, relying entirely on TS activity. The parasite genome contains a vast TS gene family, many of which are pseudogenes, reflecting its evolutionary and functional complexity.^210^ Functionally, TS facilitates host-cell invasion, vacuole escape, and cell-to-cell spread while shielding the parasite from antibody-mediated attack.^211^ ^212^ Genetic disruption of active TS genes yields attenuated, poorly invasive parasites,^213^ underscoring their essentiality.

Because TS has no human orthologue, it represents a parasite-exclusive target. However, most substrate-mimetic inhibitors — such as sialic acid analogues and transition-state mimics like DANA, or repurposed drugs zanamivir and oseltamivir — exhibit weak inhibition.^214^ ^215^ More potent inhibitors can simultaneously occupy both the donor (sialic acid) and acceptor (β-galactosyl) sites, including aryl α-aminophosphonates,^216^ triazole-based sialylmimetics,^217^ and divalent lactosides.^218^ ^219^ Additional promising candidates include benzoic acid derivatives,^220^ ^221^ phthaloyl analogues,^222^ and benzopyrazines with multi-target activity.^223^ Virtual screening has also identified hits such as ZINC13359679, ZINC02576132, and sulfasalazine, which inhibit TS *in vitro* and *in vivo*.^224^ ^225^ ^226^ Despite the redundancy of TS isoforms and its high catalytic efficiency posing challenges for drug design, its absence in humans and genetic validation continue to position TS as a prime target for anti-CD therapy and immunointervention.

## GP63 metalloprotease

The zinc-dependent metalloprotease GP63 is another major virulence determinant conserved across trypanosomatids.^227^ In *T. cruzi*, the GP63 gene family comprises eleven distinct groups, most unique to the genus, and displays complex, stage-specific expression patterns.^228^ GP63 likely contributes to immune evasion and tissue dissemination. Recent computational analyses revealed that N-aryl-1,10-phenanthroline-2-amines act as potent and non-toxic inhibitors of TcGP63, highlighting it as a promising target for structure-guided drug discovery.^229^

## Prolyl oligopeptidase (POPTc80)

The serine protease POPTc80 (prolyl oligopeptidase) is a key virulence factor that degrades large extracellular matrix components, including collagen and fibronectin, facilitating tissue migration and invasion of nonphagocytic cells.^230^ Structure-based virtual screening identified competitive POPTc80 inhibitors that effectively blocked host-cell invasion *in vitro*, confirming its role as a virulence-associated enzyme.^230^

## Prodrug activation - NTR-1

NTR-1 is a mitochondrial FMN-dependent enzyme that catalyses the two-electron reduction of nitroaromatic compounds. In *T. cruzi*, it is essential for the activation of the frontline drugs BZ and NFX, generating reactive metabolites that damage DNA, lipids, and disrupt redox balance, leading to parasite death.^49^ ^231^ Genetic studies confirm its importance: loss or downregulation of *Tc*NTR-I confers resistance, while overexpression increases sensitivity to nitro drugs.^231^ ^232^ *Tc*NTR-I is now being explored in drug discovery programs, with new nitroaromatic scaffolds (*e.g.*, nitroimidazopyrans, fexinidazole derivatives) under evaluation for selective activation.

A library of aziridinyl benzoquinones was screened and several quinones proved to be highly efficient NTR substrates, with catalytic efficiencies up to 100-fold greater than BZ or NFX.^48^ These findings highlight that the biochemical properties of trypanosomal NTRs can be harnessed for rational drug design, with quinone-based scaffolds emerging as promising trypanocidal agents.

## Disrupting cell signalling

Phosphorylation is a key regulatory mechanism governing protein activity and signalling. This reversible process is controlled by kinases and phosphatases, which together orchestrate adaptive responses to environmental cues. In *T. cruzi*, phosphoproteomic profiling during nutritional stress-induced metacyclogenesis identified 4205 protein groups, 3643 phosphopeptides, and 4846 distinct phosphorylation sites, underscoring the complexity of the parasite's signalling network.^233^ Kinases represent ~2% of the *T. cruzi* genome, forming a unique kinome that includes both eukaryotic (ePK) and atypical (aPK) protein kinases involved in essential and divergent cellular processes.^234^

A defining feature of kinetoplastid signalling is the use of kinases to sense nutrient availability and drive differentiation. A key example is *Tc*K2, a transmembrane kinase localised in endosomal nutrient reservoirs. *Tc*K2 phosphorylates eIF2α under heme deprivation, reducing translation and promoting metacyclogenesis.^235^ *Tc*K2 knockout parasites fail to differentiate properly and accumulate heme, resulting in oxidative stress. Pharmacological inhibition with Dasatinib and PF-477736 suppresses parasite proliferation, highlighting TcK2 as a promising drug target.^236^ In addition, SRPIN340 acts as a broad-spectrum kinase inhibitor in *T. cruzi*.^237^ To target heme regulatory pathways is particularly attractive since the parasite depends entirely on host-derived heme and must finely control intracellular levels to prevent toxicity.^100^

Another stress-related kinase, *Tc*K1, is a GCN2-like enzyme that mediates adaptation to nutrient limitation independently of eIF2α phosphorylation. TcK1 deletion results in increased polysome formation, reduced RNA granule assembly, enhanced metacyclogenesis, and accelerated replication in host cells.^238^

Other essential signalling components include Akt, GSK-3, and CC2CP kinases. The GSK-3 homolog, essential for kinetoplastid viability, presents structural differences from human GSK-3 that allow for selective drug design.^239^ TcAkt kinases regulate growth, proliferation, and apoptosis; inhibition strategies focusing on the PH domain PIP-binding site may provide species-selective therapeutic approaches.^240^ The cyclic nucleotide-binding/C2-domain protein TcCC2CP, involved in cAMP and Ca²⁺ signalling, is also essential — its heterozygous deletion reduces parasite growth, confirming its critical role.^241^

Collectively, these findings highlight kinase signalling networks as central regulators of nutrient sensing, stress adaptation, and differentiation in *T. cruzi*. Their evolutionary divergence from human counterparts establishes them as an expanding and highly promising therapeutic landscape for anti-CD drug discovery.

## Other targets

Carbonic anhydrases (CAs) represent a promising yet challenging class of drug targets for CD. These zinc metalloenzymes catalyse the conversion of CO2 and water into bicarbonate and protons, a critical reaction for physiological processes like pH regulation and ion balance. In *T. cruzi*, inhibition of the parasite's CA disrupts ion exchange, severely impairing metacyclogenesis and growth. The major therapeutic challenge is achieving selectivity for *Tc*CA over its highly similar human orthologs.^242^ While *Tc*CA is susceptible to sulfonamide inhibitors that bind the active-site zinc ion, these compounds often show poor *in vivo* efficacy due to limited membrane permeability. This has motivated the search for alternative chemotypes, including benzoxaboroles, anionic inhibitors (along with sulfamide, sulfamic acid, and phenylboronic/arsonic acids), hydroxamates, thiols, organoselenium compounds, and sodium cyclamate.^242^ ^243^ Furthermore, recent work combining virtual screening with experimental validation has identified nitrofurantoin and hydrazone-based compounds as potential *Tc*CA inhibitors.^244^

The lignan compound (-)-hinokinin (HNK) exhibits anti-*T. cruzi* activity. Its mechanism of action is proposed to be the inhibition of tubulin assembly, based on its structural similarity to (-)-cubebin. Molecular docking studies suggest HNK binds to both α- and β-tubulin residues, disrupting the formation of microtubules. As microtubules are essential for the parasite's cytoskeleton, this disruption leads to parasite death.^245^

## DNDi's research on compounds for CD: past efforts and current developments

Over the years, The Drug for Neglected Diseases initiative (DNDi) has explored a broad range of compounds in its quest to improve CD treatment — ranging from repurposed agents to entirely new chemical entities. DNDi is advancing its drug discovery pipeline for CD through two synergistic strategies: hit-to-lead development and open science collaborations. Through ongoing hit-to-lead alliances, DNDi is optimising new chemical entities — including outputs from HTS campaigns and medicinal chemistry programs — to accelerate the progression of candidate compounds. In parallel, DNDi is expanding the chemical diversity of its pipeline through natural product screening initiatives.^246^

## Past investigations

Azole Derivatives (*e.g.*, posaconazole, ravuconazole prodrug E1224, VNI): These antifungals demonstrated potent *in vitro* activity. In animal models, posaconazole achieved curing effects in both acute and chronic phases (murine models), and VNI — targeting CYP51 — showed complete cure in mice with excellent pharmacokinetic and safety profiles.^84^ However, results were inconsistent across different *T. cruzi* strains, and clinical translation was disappointing.^86^ E1224 (fosravuconazole, prodrug of ravuconazole) advanced to clinical studies but did not result in successful long-term cure and is no longer pursued. DNDi's proof-of-concept trial in adults with chronic indeterminate CD showed transient parasite suppression with E1224 monotherapy and sustained responses with BZ, supporting the strategy of shorter BZ regimens and selective combinations rather than azole monotherapy.^87^

Fexinidazole was elected from a large-scale screening of nitroimidazoles, fexinidazole proceeded to Phase II clinical trials in patients with chronic indeterminate CD. While the drug was well tolerated at lower, short-duration regimens, the efficacy was insufficient to match the standard of care.^247^ Consequently, DNDi halted its standalone development, although exploration of combination therapies or niche indications (*e.g.*, immunocompromised populations) remains under review.

The BENEFIT trial (Brazil) was a study designed to evaluate whether BZ could reduce clinical outcomes in patients with chronic Chagas' cardiomyopathy. The results demonstrated that while BZ reduced parasitaemia in patients with established cardiomyopathy, it did not prevent disease progression over five years.^248^

The MULTIBENZ trial evaluated whether alternative BZ regimens could achieve efficacy comparable to the standard treatment (300 mg/day for 60 days) for chronic CD while improving safety.^249^ The study demonstrated that reducing treatment duration from eight to two weeks could preserve efficacy while enhancing adherence and expanding treatment coverage. However, treatment response was markedly lower in Brazil than in other participating countries. In the control group, sustained parasitological negativity was observed in only 15% of Brazilian patients, compared with 47% in Spain, 90% in Colombia, and 85% in Argentina.^249^ The authors attributed this disparity to intrinsic parasite-related factors, consistent with previous reports of reduced treatment efficacy in Central Brazil, the region from which the Brazilian patients originated.^249^

BZ remains the standard of care and serves as the active comparator in most modern clinical trials. DNDi's BENDITA Phase 2 trial in Bolivia showed that shorter BZ regimens (including two- and four-week courses) achieved high rates of sustained quantitative polymerase chain reaction (qPCR) negativity at six months with improved tolerability versus the traditional eight-week course; these results informed ongoing work to optimise duration and dosing.^250^

## Current and emerging efforts

The ongoing NuestroBen trial (Argentina) is a phase III, randomised, multicentre study designed to determine if shorter (two- and four-week) BZ regimens are equal or better in efficacy than the standard eight-week course for patients with indeterminate or mild cardiac CD. Another objective is to assess whether these short regimens improve drug tolerability and treatment adherence. The trial is estimated to be completed by September 2026.^251^

*Early clinical development* - AN2-502998 (benzoxaborole; CPSF3 pathway). In 2025 DNDi and AN2 Therapeutics announced a collaboration; Phase I start-up is underway with completion targeted in late 2025 and a Phase II proof-of-concept planned for 2026. This candidate builds on benzoxaborole validation across kinetoplastids and the emerging CPSF3 mechanism in *T. cruzi*.^252^ ^253^

*Preclinical leads* - AN15368 (benzoxaborole prodrug; CPSF3 mechanism) demonstrated uniform cures in naturally infected non-human primates and broad activity across genetically diverse *T. cruzi* lineages, providing rare translational evidence for a new class and de-risking clinical entry.^181^

New Chemical Series (*e.g.*, UW Series, AN2-502998, Series-5824): DNDi has advanced novel scaffolds with promising preclinical performance — including compounds showing sterile cure in chronic murine models — and continues to optimise these leads in collaboration with global partners.^246^

## Challenges in CD treatment

Developing safe, effective, and selective drugs against CD has been challenging, despite the fact *T. cruzi* being a biologically unique eukaryotic organism. The development process is hindered by a complex array of factors, including parasite genetic and phenotypic diversity, limited validated molecular targets, drug resistance mechanisms, the presence of dormant forms, an incipient search for host-directed therapies, translational gaps between preclinical and clinical studies, a lack of reliable biomarkers of cure, and the intricate interplay between host, parasite, and the immune system.

## Parasite genetic and phenotypic diversity

*T. cruzi* exhibits remarkable genetic and phenotypic heterogeneity, currently classified into at least seven discrete typing units (DTUs, TcI-TcVI and TcBat), which differ in geographic distribution, tissue tropism, virulence, metabolic pathways, and drug susceptibility.^47^ ^254^ ^255^ This variability complicates the identification of universally effective compounds, as hits active against one lineage may show reduced efficacy in others. In addition to strain-specific differences, stage-specific forms of the parasite — including epimastigotes, replicative amastigotes, bloodstream trypomastigotes, and dormant non-replicative amastigotes pose further challenges for drug activity across the life cycle. Such complexity underscores the importance of validating candidate compounds across multiple DTUs and developmental stages to ensure broad-spectrum efficacy and minimise the risk of strain- or stage-specific therapeutic failure.^24^

## Drug resistance mechanisms

The natural resistance of some *T. cruzi* strains to BZ and NFX presents a major therapeutic challenge.^57^ Resistance to BZ and NTX, the two approved drugs for CD, arises through both nitroreductase (TcNTR-1)-dependent and independent mechanisms. Mutations or loss of TcNTR-1 reduce prodrug activation, while alternative adaptations such as enhanced antioxidant defences, increased DNA repair, efflux pump overexpression, and redox alterations further protect the parasite from drug-induced damage.^50^ ^62^ Deletion of specific metabolic enzymes like prostaglandin F2 synthase is another important mechanism.^42^ ^256^ ^257^ This multifactorial resistance, observed in diverse *T. cruzi* strains, underscores the parasite's capacity to adapt under drug pressure and highlights the urgent need for compounds with novel mechanisms of action and combination therapies to overcome cross-resistance.

## Persisters and dormant forms

The existence of dormant, metabolically inactive and non-replicative amastigotes of *T. cruzi* represents a major barrier to the effective treatment of CD. These intracellular, non-motile forms can spontaneously enter a state of arrested replication, independent of drug exposure, allowing them to tolerate BZ and NTX and persist within host cells despite prolonged treatment or high drug concentrations.^58^ ^258^ ^259^ ^260^ Upon drug withdrawal, dormant parasites can resume replication, leading to recrudescence and therapeutic failure. Dormancy has been linked to DNA damage responses and repair pathways.^41^

The persistence of these forms helps explain frequent treatment failures and underscores the urgent need to elucidate the molecular mechanisms governing dormancy. Developing therapeutic strategies capable of targeting both replicative and dormant populations is therefore essential to achieve parasitological cure and improve clinical outcomes in CD.^258^ ^259^

## Addressing target validation and translational gaps

Although several enzymes and pathways have been proposed as potential drug targets — such as CYP51, cruzipain, and trypanothione reductase — few have been robustly validated *in vivo*.^30^ ^261^ Target essentiality is often strain- or stage-dependent, and redundancy in metabolic pathways can diminish the efficacy of single-target inhibition. Moreover, difficulties in genetic manipulation of *T. cruzi* historically delayed target validation, though CRISPR/Cas9 has recently improved this process.

Phenotypic hits with strong *in vitro* activity often fail *in vivo* due to poor pharmacokinetics, toxicity, or lack of efficacy in chronic infection models.^26^ Even promising clinical candidates, such as posaconazole and ravuconazole (azole CYP51 inhibitors), did not achieve sterile cure in chronic patients despite potent preclinical activity.^86^ This translational gap emphasises the need for standardised, predictive models and endpoints. A promising strategic avenue involves the development of molecular hybrids or synergistic drug combinations that integrate the most potent known inhibitors to enhance therapeutic efficacy.^110^

## Incipient search for “antichagasic therapy”

Past drug discovery for CD prioritised finding compounds that directly kill the parasite. Current research is broadening to pursue a complete “antichagasic therapy,” which includes prophylactic and therapeutic vaccines, host-directed therapies (modulating the human host's response) and drugs designed to treat the specific cardiomyopathy that CD causes.^26^

## Lack of reliable biomarkers of cure

One of the most critical obstacles in CD drug development is the lack of validated biomarkers capable of reliably monitoring short-term treatment response. Currently, serological markers remain positive for years or even decades after therapy, making them unsuitable for assessing patient cure within the timeframe of clinical trials.^24^ In contrast, non-conventional serological assays that detect lytic antibodies^262^ or their analogues, such as tests measuring IgG anti-live trypomastigote antibodies,^263^ offer the advantage of enabling an earlier determination of parasitological cure in *T. cruzi* infection when negative. Although qPCR is frequently used as a surrogate endpoint, persistent negativity does not necessarily equate to sterilising cure, as parasites may persist at levels below detection limits.^26^ ^264^ This diagnostic gap delays the evaluation of therapeutic efficacy, complicates the validation of preclinical models, and ultimately slows the development and approval of new treatments.^22^ The establishment of robust, standardised, and predictive biomarkers of cure is therefore essential to accelerate drug discovery and improve clinical trial design in CD.^24^ ^26^

## Host-parasite and immune system interactions

The outcome of infection and treatment is strongly influenced by host immune responses, which vary widely across individuals. Drugs effective in immunocompetent models may fail in immunosuppressed conditions. Moreover, compounds must penetrate host tissues (*e.g.*, cardiac and gastrointestinal muscle, bladder, adipose tissue, among others) where parasites persist in protected niches, a barrier for achieving sterilising cure.^24^

## Economic and logistical constraints

The term “Neglected Tropical Diseases” (NTDs) was first introduced in 2003, and since 2005, the World Health Organisation (WHO) has classified CD as part of this group.^1^ Like other NTDs, CD poses major challenges because it is deeply entwined with poverty and social inequality.

Economic and access barriers remain central obstacles to disease control. These include the unequal distribution of medical infrastructure that disadvantages rural populations, high treatment costs, limited capacity to manage or reverse cardiac complications, and the lack of reliable prognostic tools.^3^ ^5^ ^7^ Even basic access to existing medication and its continuous, reliable production are ongoing challenges.^7^ ^265^ ^266^ Fewer than 10% of Chagas patients are diagnosed in time, and just 1% receive adequate treatment.^2^ ^4^ ^6^

Beyond its social determinants, disease outcomes and treatment responses are influenced by both parasite variability and patient-specific genetic and physiological factors, emphasising the potential of personalised medicine in CD management.^267^ ^268^ However, when basic access to therapy is still uncertain, precision health remains an aspirational goal — technological progress alone cannot overcome the structural inequities sustaining disease burden.

As a NTD, CD suffers from limited pharmaceutical investment. Many promising compounds stall in early discovery due to resource limitations. The cost of long-term clinical trials in endemic areas further slows progress. Public-private partnerships and nonprofit consortia (*e.g.*, DNDi) play crucial roles, but funding gaps persist.^28^

## Concluding remarks

Overall, studies on molecular targets in *T. cruzi* have provided significant advances in identifying key proteins associated with drug resistance, redox detoxification, cellular homeostasis, and differentiation. Despite this progress, many investigations remain limited to *in vitro* approaches, underscoring the need for validation in animal models and more comprehensive functional studies. The inherent metabolic redundancy of the parasite further challenges the development of effective single-target strategies, highlighting the importance of combinatorial or multi-target approaches. Moreover, the limited functional annotation of *T. cruzi* proteins suggests that additional efforts in systems biology, comparative genomics, and integrative omics analyses will be essential to expand the repertoire of validated targets.

The exploration of molecular targets in *T. cruzi* represents a promising avenue to overcome the limitations of current chemotherapy. Transporters, redox enzymes, and regulators of translation and stress response emerge as particularly relevant candidates, given their involvement in BZ resistance and parasite adaptation to environmental pressures. Future progress will likely depend on the integration of high-throughput omics data, CRISPR/Cas9-based gene editing, and compound screening assays, which together may accelerate target validation and drug discovery. Importantly, continuous refinement of these approaches may enable the identification of novel therapeutic strategies, ultimately contributing to more effective and less toxic treatments for CD.

While the development of novel therapeutics is crucial, effectively addressing CD requires confronting significant non-scientific challenges. Profound economic and access barriers impede effective disease management. These systemic hurdles must be considered as critically as the search for new technologies, so that any new treatment can reach the vulnerable populations who need it most.

## SUPPLEMENTARY MATERIALS

Supplementary material

## Data Availability

No new primary data was generated for this review. All data discussed are derived from the published studies cited in the reference list.
