# Supplementary material for: Advances and challenges in the search for new treatments for Chagas disease
Source: Mem Inst Oswaldo Cruz. 2026 Jul 10;121(Suppl 2):e250299. doi: 10.1590/0074-02760250299 (PMC13361135; doi:10.1590/0074-02760250299)
Supplement: Supplementary material [file 1678-8060-mioc-121-s2-e250299-s1.pdf]

TABLE

Summary of key metabolic pathways / drug targets identified in *Trypanosoma cruzi*, along with their corresponding known inhibitors

| Metabolic pathways/drug targets | Inhibitors | References |
|---|---|---|
| **Ergosterol biosynthesis** | | |
| CYP51 | azole-based antifungals | (84,85) |
| HMG-CoA-R | lovastatin and simvastatin | (91) |
| Membrane transporters | | |
| Calcium channels | Gaba, Amlod, Pinav, and amiodarone derivatives | (97,98) |
| HRG | Bleomycin, pyrvinium pamoate, lauryl gallate and others | (99,100) |
| **Glycolytic pathway** | | |
| GlcK | 3-nitro-2-phenyl-2H-chromene analogs and CBZ-GlcN | (105,106) |
| PFK | ML251, apyrrolopyridine derivatives (CTCB series) and CTCB405 | (107,108,109) |
| TIM | Thiazole, benzothiazole, and benzimidazole derivatives | (110) |
| GAPDH | Anacardic acid derivatives, NO donors cis-[Ru(NO)(bpy)2L]Xn and adenosine derivatives | (111,112,113113) |
| **Pentose phosphate pathway (PPP)** | | |
| 6PGDH | sulfoxide derivates and Hydroxamate and amide derivatives of D-erythronic acid | (115) |
| RPI-B | Haloacetamide analogs | (114) |
| RPE | Haloacetamide analogs | (114) |
| **Cytochrome B** | | |
| Cytb | GNF7686, antimycin A, Pyrazolopyrimidinone and quinazolinedione derivatives | (119,120) |
| **Nucleotide biosynthesis** | | |
| DHFR-TS | Methotrexate (MTX), nolatrexed and antifolate derivatives | (33,129,130,131) |
| DHOD | orotate analogues, thiazolidines, arylideneketones and flavonoids | (33,110,129,130,131) |
| HGPRT | Allopurinol and other nucleoside analogues | (133-142) |
| **Redox homeostasis mechanisms** | | |
| FeSODs | Phthalazines, benzo[g]phthalazines, macrocycles, abietic acid derivatives, dithiocarbamates, and Mannich-base arylamines—with optimized imidazole and benzimidazole- and pyrrole-benzo[g] phthalazine analogues | (145,146) |
| GAL | allylpolyalkoxybenzenes group (APABs) | (148) |
| TR | Phenothiazine-Based derivatives, sesquiterpene lactones, polyamines, quinones, metal complexes, Mannich bases and phenothiazine derivatives | (110,150,152) |
| TryS | Paullone-derived inhibitors, 4,5-dihydroazepino[4,5-b]indol-2(1H,3H,6H)-one derivatives and others | (153,154) |
| SpdSyn | 2-[(4,6-dihydroxy-1,3,5-triazin-2-yl)amino]-4H-1,3-benzothiazin-4-one, trans-4-methylcyclohexylamine and others | (155,156) |
| DNA maintenance | | |
| KKT | Stausporine, hypothemicyn, compounds 1 and 2 | (160) |
| Topo3α | Thiosemicarbazones, anthracycline and cyanotriazole-based compounds | (91,162) |
| **Epigenetics and epitranscriptomics** | | |
| KATs | Curcumin and Triptolide | (164,165,166) |
| KDACs | trichostatin A (TSA) and hydroxamate- or benzamide-based small molecules | (166,167) |
| BRDs | A1B4, iBET151,1,3,4-oxadiazoles, SGC-CBP30, bromosporine, GSK2801 and I-BRD9 | (169,170) |
| **mRNA biogenesis and turnover** | | |
| TBP | DB00890 | (178) |
| CPSF3 | AN15368 | (181) |
| **Translation machinery** | | |
| eIF4A | hippuristanol, rocaglates and pateamine A | (188) |

↳

| Metabolic pathways/drug targets | Inhibitors | References |
|---|---|---|
| **Protein homeostasis and metabolic dependencies** | | |
| LAPTc | Bestatin, compound 4 (C19H15N3O3S) and others | (190,191) |
| TDH | TCMDC-143160 | (192) |
| KRS1 | Quinazoline-based inhibitors | (193) |
| Hsp90/Hsp83 | Geldanamycin and 17-DMAG | (194,195) |
| Sir2rp1 | Benznidazole-derived inhibitors (BNIPs) | (196,197) |
| Proteasome | GNF6702, GSK3494245, LCQFTC11, pyridazinone analogues and others | (30,199,200,202) |
| **Virulence factors** | | |
| CZP | thio- and semicarbazones, imidazoles, benzimidazoles, coumarins, oxadiazoles, thiazoles, quinoxalines, triazoles, pyrimidines, hydrazones, benzoyl thioureas, and hydroxymethyl ketones | (30,110,204,205,206,207,208) |
| TS | aryl α-aminophosphonates, triazole-based sialylmimetics, divalent lactosides, benzoic acid derivatives, phthaloyl analogues, benzopyrazines, ZINC13359679, ZINC02576132, and sulfasalazine | (214-226) |
| GP63 | N-aryl-1,10-phenanthroline-2-amines | (229) |
| POPTc80 | LC-44, LC-45, LC-46, LC-50, LC-53, and LC-55 | (230) |
| **Prodrug activation** | | |
| NTR1 | Aziridinyl benzoquinones series | (48) |
| **Cell signalling** | | |
| K2 | Dasatinib, PF-477736 and SRPIN340 | (236,237) |
| **Other targets** | | |
| CAs | Acetazolamide and sulfonamide derivatives | (242,243,244) |

BDF2: Bromodomain-containing factor 2; BRDs: Bromodomain proteins; CAs: Carbonic anhydrases; CPSF3: Cleavage and polyadenylation specificity factor 3; CZP: Cruzipain; CYP51: Sterol 14α-demethylase (cytochrome P450 51); Cytochrome B / Cytb: Cytochrome b, mitochondrial respiratory chain componente; DHFR-TS: Dihydrofolate reductase-thymidylate synthase; DHOD: Dihydroorotate dehydrogenase; eIF4A: Eukaryotic translation initiation factor 4A; FeSODs: Iron superoxide dismutases; GAL: Galactonolactone oxidase; GAPDH: Glyceraldehyde-3-phosphate dehydrogenase; GlcK: Glucokinase; GP63: Glycoprotein 63; HGPRT: Hypoxanthine-guanine phosphoribosyltransferase; HMG-CoA-R: 3-Hydroxy-3-methylglutaryl-coenzyme A reductase; HRG: Heme-responsive gene protein; Hsp90/Hsp83: Heat shock protein 90 / 83; K2: Protein kinase 2; KATs: Lysine acetyltransferases; KDACs: Lysine deacetylases; KKT: Kinetoplastid kinetochore proteins; KRS1: Lysyl-tRNA synthetase 1; LAPTc: Leucyl-aminopeptidase from *T. cruzi;* NTR1 -nitroreductase Type I; PFK: Phosphofructokinase; 6PGDH: 6-Phosphogluconate dehydrogenase; POPTc80: Oligopeptidase Tc80 (prolyl oligopeptidase); RPE: Ribulose-5-phosphate epimerase; RPI-B: Ribose-5-phosphate isomerase B; Sir2rp1: Cytoplasmic sirtuin 2-related protein 1; SpdSyn: Spermidine synthase; TBP: TATA-box binding protein; TDH: L-Threonine dehydrogenase; TcA: *T. cruzi* trans-sialidase antigen A; TcC: *T. cruzi* trans-sialidase antigen C; TIM: Triosephosphate isomerase; Topo3α: DNA topoisomerase III alpha; TR: Trypanothione reductase; TryS: Trypanothione synthetase; TS: Trans-sialidase.
