# Supplementary material for: Advances and challenges in the search for new treatments for Chagas disease
Source: Mem Inst Oswaldo Cruz. 2026 Jul 10;121(Suppl 2):e250299. doi: 10.1590/0074-02760250299 (PMC13361135; doi:10.1590/0074-02760250299)
Supplement: Peer review history

# Reviewer report: FIRST REVIEW ROUND - REVIEWERS COMMENTS

## REVIEWER #1

The manuscript titled “Advances and Challenges in the Search for New Treatments for Chagas Disease” by Santi et al. presents an elegant and relevant review of the literature in the context of research into new treatments for Chagas disease. The manuscript is exceptionally well-written, balanced, and informative, addressing the most significant advances and challenges in the research of new therapeutic molecules and exploring potential targets for the development of new drugs.

Some minor corrections are necessary:

On page 25, line 48, the term *Trypanosoma cruzi* should be italicized.

Please review the text for the terms *in vitro* and *in vivo*, which should also be italicized throughout.

## REVIEWER #2

The review presents a lengthy and detailed description of “Advances and Challenges in the Search for New Treatments for Chagas Disease.” The English writing style needs improvement.

Also, some suggestions need to be considered as follows:

1. Page 3 lines 34-36: Also include a brief discussion of the relevant dissemination of treatment for infected individuals, given that less than 1% of them receive treatment with the available therapies.

2. Page 5 line 33: Also include, the performance in *in vivo* tests.

3. Page 5 line 53: Include “…and as combined with reference drugs for CD”. Include information and references.

4. Page 6 line 38: I would rather describe that “allowing, in earlier drug discovery steps, to integrate information from both ligands and targets to predict bioactivity or binding affinity.

5. Page 6 Lines 49-53: Too long and confusing sentences. Rewrite.

6. Page 7 line 3: correct: “central mode”

7. Page 7 lines 3-13: Too long sentences. Rephrase it. Also, the bulk of recent data suggest that in *T. cruzi* the resistance to benznidazole may by a multigenic trait. Include in your discussion, the role of nitroreductase I in the resistance mechanisms.

8. Page 8 line 12: correct as follows: “ among others) (50,58).”

9. Page 8 lines 54-55: Revise the sentence: Is biosynthesis of ergosterol?

10. Page 9 lines 17-19: Include a brief discussion of the possible reasons for the failure of posaconazole and E1224 in clinical trials and the discrepancy between preclinical and clinical findings for both azoles, such as the mechanism of action, the drug exposure achieved in animal models compared to humans, and the low sequence identification (≈25%) of trypanosomatids and fungi CYP51.

11. Page 13 line 27: Several studies describe the promising effects of various nucleoside analogues (such as 7-deazapurines, among others) *in vitro* and *in vivo*, justifying their further investigations as therapeutic agents for CD. I suggest including some of these articles.

12. Page 24 lines 28-29: Review the data concerning Brazilian chronic patients who presented high rates of short-term therapeutic failure with the administration of benzimidazoles and briefly include other clinical trials, such as NuestroBen. In lines 34-39: Move to subitem 6.1

13. Page 25 line 29: Replace “specific” by “selective”. In line 48, *Trypanosoma cruzi* in italic.

14. Page 28 line 35: The infection can be sustained in other tissues as well including bladder, adipose tissue, among others.

## AUTHORS' RESPONSE TO THE REVIEWERS

Dear Editor and Reviewers,

Thank you for the opportunity to revise our manuscript, “Advances and Challenges in the Search for New Treatments for Chagas Disease”. We are grateful to the reviewers for their thorough reading, careful evaluation, and insightful feedback. We have incorporated all the suggestions, and the revised manuscript now reflects the required changes. We believe the changes have significantly improved the quality and clarity of the manuscript and hope it is now deemed suitable for publication. Our responses to each specific comment are provided in the section below.

Thank you for your consideration.

Sincerely,

Best regards,

Santi, Alvarenga, and Murta

## REVIEWER: 1

Reviewer comments: The manuscript titled “Advances and Challenges in the Search for New Treatments for Chagas Disease” by Santi et al. presents an elegant and relevant review of the literature in the context of research into new treatments for Chagas disease. The manuscript is exceptionally well-written, balanced, and informative, addressing the most significant advances and challenges in the research of new therapeutic molecules and exploring potential targets for the development of new drugs.

Some minor corrections are necessary:

On page 25, line 48, the term *Trypanosoma cruzi* should be italicized.

Response: Thank you for pointing out this error. The term *Trypanosoma cruzi* has been italicized as suggested.

“*T. cruzi* exhibits remarkable genetic and phenotypic heterogeneity, currently classified into at least seven discrete typing units (DTUs, TcI–TcVI and TcBat), which differ in geographic distribution, tissue tropism, virulence, metabolic pathways, and drug susceptibility (47,243,244).”

Please review the text for the terms *in vitro* and *in vivo*, which should also be italicized throughout.

Response: We appreciate you highlighting this issue, all the text was reviewed.

## REVIEWER: 2

Reviewer comments: The review presents a lengthy and detailed description of “Advances and Challenges in the Search for New Treatments for Chagas Disease.” The English writing style needs improvement.

Response: We thank the Reviewer for the comment regarding the English writing style. In response, the entire manuscript has been carefully reviewed and revised to improve clarity, consistency, and overall language quality. Although Reviewer 1 considered the manuscript to be well written, we have implemented additional linguistic refinements to further enhance the text.

Also, some suggestions need to be considered as follows:

1. Page 3 lines 34-36: Also include a brief discussion of the relevant dissemination of treatment for infected individuals, given that less than 1% of them receive treatment with the available therapies.

Response: We appreciate you highlighting this issue. We have updated that section:

“The emergence of drug-resistant *T. cruzi* strains; high medical costs, and recurrent drug shortages further complicate disease management. Moreover, limited healthcare infrastructure outside urban centers; the fact that few patients are diagnosed in time and even fewer receive adequate treatment; difficulties in rehabilitating Chagas-related cardiomyopathy; and the lack of reliable prognostic biomarkers remain major obstacles to effective control and patient care (2–7).”

Instead of adding this information only to the introduction, we decided to also improve the item 7.8 (Economic and Logistical Constraints):

“Economic and access barriers remain central obstacles to disease control. These include the unequal distribution of medical infrastructure that disadvantages rural populations, high treatment costs, limited capacity to manage or reverse cardiac complications, and the lack of reliable prognostic tools (3,5,7). Even basic access to existing medication and its continuous, reliable production are ongoing challenges (7,254,255). Fewer than 10% of Chagas patients are diagnosed in time, and just 1% receive adequate treatment (2,4,6).”

2. Page 5 line 33: Also include, the performance in *in vivo* tests.

Response: Thank you for the suggestion. We have revised this section accordingly:

“Nevertheless, translation has been challenging, as demonstrated by the clinical failure of azole antifungals such as posaconazole and ravuconazole, which showed strong *in vitro* and *in vivo* efficacy against CYP51 but failed to outperform benznidazole in patients (26,31). This discrepancy highlights the need for a critical re-evaluation of preclinical models and protocols (31). For a detailed discussion, see Sections 3.6 (*in vivo* studies), and 5.1 (Inhibiting the Ergosterol Biosynthesis Pathway).”

We have also added a missing section, 3.6. *In Vivo* Studies:

“Promising drug candidates identified through *in vitro* screening are subsequently advanced to *in vivo* models, where their safety and ability to significantly reduce parasitemia are evaluated. This translational step, however, remains challenging due to the lack of standardized animal models. The use of different parasite strains, host species, and infection routes to address specific aspects of the disease complicates cross-study comparisons and slows the drug discovery process (20). Given that no single model fully recapitulates Chagas disease, a more realistic and effective strategy is the development of multiple robust and standardized models rather than the pursuit of a single ideal system (20).

Murine models are the most widely employed. According to the protocol proposed by Romanha and colleagues (24,46), murine drug screening follows a multi-stage approach in which promising compounds are first evaluated in acute infection models to assess efficacy and define optimal dosing. Candidates that show favorable profiles are then tested in chronic infection models, where their ability to prevent parasite relapse following immunosuppression is assessed, thereby mimicking the clinical challenge of eliminating tissue-resident parasites. In light of current knowledge regarding the dynamic and tissue-specific colonization of *T. cruzi* during chronic infection, the use of chronic models is now strongly recommended for drug evaluation (24).

Canine and non-human primate models also offer important translational value for human Chagas disease. Dogs naturally develop chronic cardiac manifestations similar to those observed in humans, while non-human primates are physiologically the closest model to humans. Nevertheless, their use is constrained by high costs, logistical complexity, and ethical considerations (20).

Finally, the use of genetically modified parasites expressing luciferase or fluorescent reporters such as tdTomato represents a major advance in drug testing. These systems enable not only high-throughput *in vitro* screening but also non-invasive, longitudinal monitoring of infection dynamics *in vivo* (21,47)”.

3. Page 5 line 53: Include “…and as combined with reference drugs for CD”.

Include information and references.

Response: Thank you for raising this point. We have revised this section accordingly:

“Several repositioned antifungals, including posaconazole and ravuconazole, advanced to clinical trials but ultimately failed as monotherapies, and as combined with reference drugs for CD, due to insufficient efficacy against chronic infection (26,31) (as referenced in Section 3.2). Nonetheless, repositioning remains attractive when pursued in combination with phenotypic assays and rational selection, offering a rapid entry point for candidate prioritization and providing scaffolds for medicinal chemistry optimization.”

4. Page 6 line 38: I would rather describe that “allowing, in earlier drug discovery steps, to integrate information from both ligands and targets to predict bioactivity or binding affinity.

Response: Thank you for your suggestion, which we have now incorporated into the text:

“In parallel, machine learning and artificial intelligence (AI) approaches are increasingly applied to predict biological activity, toxicity, and ADMET (absorption, distribution, metabolism, excretion, toxicity) parameters, thereby allowing, in earlier drug discovery steps, to integrate information from both ligands and targets to predict bioactivity or binding affinity (26).”

5. Page 6 Lines 49-53: Too long and confusing sentences. Rewrite.

Response: Thank you for the feedback. We have updated that entire section:

“Omics technologies provide crucial, system-wide insights into drug action and resistance in *T. cruzi*. These tools map how compounds perturb parasite biology and identify the adaptive responses that arise under pharmacological pressure. Current evidence positions benznidazole resistance in *T. cruzi* as a multigenic trait, as demonstrated by several omics studies. Proteomic profiling of resistant strains reveals upregulation of molecular chaperones and proteins involved in redox metabolism, including key antioxidant enzymes (36). Metabolomic profiling demonstrated significant remodeling of energy metabolism and lipid utilization during benznidazole treatment (37,38). Meanwhile, transcriptomic analyses reveal differential expression of genes linked to oxidative stress response and DNA repair in benznidazole-resistant *T. cruzi* strains (39–42). Collectively, these findings show that resistance in *T. cruzi* is mediated by broad metabolic and transcriptional reprogramming rather than single-gene alterations. This multigenic aspect will be explored in further detail in Section 7.2 (Drug Resistance Mechanisms).

The development of CRISPR/Cas9-based genome editing in *T. cruzi* has opened new avenues for validating drug targets and dissecting resistance mechanisms (43–45). Together with the Omics, these studies offer a foundation for rational drug development and the identification of biomarkers predictive of treatment outcomes.”

6. Page 7 line 3: correct: “central mode”

Response: Thank you. We have revised the sentence to enhance its clarity and logical flow:

“Proteomic profiling of resistant strains reveals upregulation of molecular chaperones and proteins involved in redox metabolism, including key antioxidant enzymes (36).”

7. Page 7 lines 3-13: Too long sentences. Rephrase it. Also, the bulk of recent data suggest that in *T. cruzi* the resistance to benznidazole may by a multigenic trait. Include in your discussion, the role of nitroreductase I in the resistance mechanisms.

Response: Thank you for the feedback. We have updated that section:

“Current evidence positions benznidazole resistance in *T. cruzi* as a multigenic trait, as demonstrated by several omics studies. (…) Collectively, these findings show that resistance in *T. cruzi* is mediated by broad metabolic and transcriptional reprogramming rather than single-gene alterations. This multigenic aspect will be explored in further detail in Section 7.2 (Drug Resistance Mechanisms).”

The discussion of nitroreductase I’s role is provided in Section 7.2.

8. Page 8 line 12: correct as follows: “ among others) (50,58).”

Response: Thank you for the feedback. We have updated that section:

“Combinations under investigation include antifungal agents (itraconazole, ketoconazole, posaconazole, ravuconazole, voriconazole), arylimidamides, ascorbic acid, acetylsalicylic acid, levamisole, clomipramine, fenofibrate, simvastatin, clofazimine, and benidipine, among others (56,64).”

9. Page 8 lines 54-55: Revise the sentence: Is biosynthesis of ergosterol?

Response: Thank you for the correction. We have corrected this mistake:

“The ergosterol biosynthesis pathway has been extensively explored as drug target in *T. cruzi*. Unlike human cells that produce cholesterol, trypanosomes rely on the synthesis of ergosterol for survival.”

10. Page 9 lines 17-19: Include a brief discussion of the possible reasons for the failure of posaconazole and E1224 in clinical trials and the discrepancy between preclinical and clinical findings for both azoles, such as the mechanism of action, the drug exposure achieved in animal models compared to humans, and the low sequence identification (≈25%) of trypanosomatids and fungi CYP51.

Response: Thank you for raising this point. The relevant part of the paragraph has been rewritten:

“However, clinical trials in chronic indeterminate disease using posaconazole (84) and E1224 (85) failed to achieve durable parasitological cure, likely due to their cytostatic—rather than cytocidal—effects, a shortcoming confirmed by both long-term *in vitro* assays and highly sensitive *in vivo* imaging (86,87). Furthermore, structural variations in the CYP51 enzyme across *T. cruzi* strains can result in diminished azole binding affinity (88). Collectively, these shortcomings led to the deprioritization of CYP51 as a drug target.”

11. Page 13 line 27: Several studies describe the promising effects of various nucleoside analogues (such as 7-deazapurines, among others) *in vitro* and *in vivo*, justifying their further investigations as therapeutic agents for CD. I suggest including some of these articles.

Response: We appreciate you highlighting this issue. We have updated that section: “Their essential roles in purine salvage have been exploited pharmacologically: allopurinol, a purine analog, inhibits parasite growth by acting as an alternative substrate for TcHGPRT (131). This dependency on purine salvage can also be exploited using other nucleoside analogues, including allopurinol ribonucleoside, 9-deazainosine, formycin B, 7-(4-chlorophenyl)-aminopurinol ribonucleoside, tubercidin (7-deazaadenosine), cordycepin (3′-deoxyadenosine), 7-(4-chlorophenyl)-3′-deoxy-7-deazaadenosine, 9-deazadenosines, and N6-methyltubercidin (132).”

12. Page 24 lines 28-29: Review the data concerning Brazilian chronic patients who presented high rates of short-term therapeutic failure with the administration of benzimidazoles and briefly include other clinical trials, such as NuestroBen. In lines 34-39: Move to subitem 6.1

Response: Thank you for raising this point. We have revised sections 6.1 and 6.2, accordingly:

6.1. Past Investigations

Azole Derivatives (e.g., posaconazole, ravuconazole prodrug E1224, VNI): These antifungals demonstrated potent *in vitro* activity. In animal models, posaconazole achieved curing effects in both acute and chronic phases (murine models), and VNI—targeting CYP51—showed complete cure in mice with excellent pharmacokinetic and safety profiles. However, results were inconsistent across different *T. cruzi* strains, and clinical translation was disappointing (84). E1224 (fosravuconazole, prodrug of ravuconazole) advanced to clinical studies but did not result in successful long-term cure and is no longer pursued. DNDi’s proof-of-concept trial in adults with chronic indeterminate CD showed transient parasite suppression with E1224 monotherapy and sustained responses with benznidazole, supporting the strategy of shorter BZ regimens and selective combinations rather than azole monotherapy (85).

The BENEFIT trial (Brazil) was study designed to evaluate whether BZ could reduce clinical outcomes in patients with chronic Chagas’ cardiomyopathy. The results demonstrated that while BZ reduced parasitemia in patients with established cardiomyopathy, it did not prevent disease progression over five years (237).

Fexinidazole was elected from a large-scale screening of nitroimidazoles, fexinidazole proceeded to Phase II clinical trials in patients with chronic indeterminate CD. While the drug was well tolerated at lower, short-duration regimens, the efficacy was insufficient to match the standard of care (238). Consequently, DNDi halted its standalone development, although exploration of combination therapies or niche indications (e.g., immunocompromised populations) remains under review.

6.2. Current and Emerging Efforts

BZ remains the standard of care and has been the active comparator in most modern trials. DNDi’s BENDITA Phase 2 trial in Bolivia showed that shorter BZ regimens (including 2- and 4-week courses) achieved high rates of sustained qPCR negativity at 6 months with improved tolerability versus the traditional 8-week course; these results informed ongoing work to optimize duration and dosing (239).

Similarly, the ongoing NuestroBen trial (Argentina) is a phase III, randomized, multicenter study designed to determine if shorter (2- and 4-week) BZ regimens are equal or better in efficacy than the standard 8-week course for patients with indeterminate or mild cardiac Chagas disease. Another objective is to assess whether these short regimens improve drug tolerability and treatment adherence. The trial is estimated to be completed by September 2026 (240).

13. Page 25 line 29: Replace “specific” by “selective”. In line 48, *Trypanosoma cruzi* in italic.

Response: Thank you for the correction. We have updated that section:

“Developing safe, effective, and selective drugs against CD has been challenging, despite the fact *T. cruzi* being a biologically unique eukaryotic organism.”

14. Page 28 line 35: The infection can be sustained in other tissues as well including bladder, adipose tissue, among others.

Response: Thank you for your careful review. The text has been revised accordingly:

“Drugs effective in immunocompetent models may fail in immunosuppressed conditions. Moreover, compounds must penetrate host tissues (e.g., cardiac and gastrointestinal muscle, bladder, adipose tissue, among others) where parasites persist in protected niches, a barrier for achieving sterilizing cure (24).”

# Reviewer report: REVIEWERS COMMENTS

## REVIEWER #1

The revised form of the review “Advances and Challenges in the Search for New Treatments for Chagas Disease” has clearly improved. However, few points still need to be addressed:

1. Lines 18-21: Include the references of VNI/VFV studies that are not included and cited (For VNI: Soeiro Mde N, de Souza EM, da Silva CF, Batista Dda G, Batista MM, Pavão BP, Araújo JS, Aiub CA, da Silva PB, Lionel J, Britto C, Kim K, Sulikowski G, Hargrove TY, Waterman MR, Lepesheva GI. *In vitro* and *in vivo* studies of the antiparasitic activity of sterol 14α-demethylase (CYP51) inhibitor VNI against drug-resistant strains of *Trypanosoma cruzi*. Antimicrob Agents Chemother. 2013 Sep;57(9):4151-63. doi: 10.1128/AAC.00070-13. For VFV: Guedes-da-Silva FH, Batista DDGJ, Da Silva CF, Pavão BP, Batista MM, Moreira OC, Souza LRQ, Britto C, Rachakonda G, Villalta F, Lepesheva GI, Soeiro MNC. Successful Aspects of the Coadministration of Sterol 14α-Demethylase Inhibitor VFV and Benznidazole in Experimental Mouse Models of Chagas Disease Caused by the Drug-Resistant Strain of *Trypanosoma cruzi*. ACS Infect Dis. 2019 Mar 8;5(3):365-371. doi: 10.1021/acsinfecdis.8b00253.

2. Lines 34-44: Nwoke ET AL., 2024 is a review. Include some references from the cited targets (e.g. cordycepin (3′-deoxyadenosine), 7-(4-chlorophenyl)-3′-deoxy-7 deazaadenosine, 9-deazadenosines, and N6-methyltubercidin among others).

3. Include in item 5.2 (and not in item 6.2 as described in the response letter) the information regarding Brazilian chronic patients who presented high rates of therapeutic failure (not sustained parasitological negativity by qPCR) with the administration of benznidazole in the short-term of drug administration (2 weeks): see at Bosh-Nicolau et al., 2024 LANCET 24:386:394. “…an overall low treatment response in the Brazilian population compared with the other countries . In the control group in the ITT population, the probability of sustained parasitological negativity was 15% (95% CI 5–38) in Brazil compared with 47% (22–73) in Spain, 90% (58–98) in Colombia, and 85% (59–96) in Argentina”.

4. Revised the references accordingly with the revised MS.

## REVIEWER #2

The authors adequately addressed all points raised by the reviewers, making the manuscript more accurate. The manuscript has high scientific relevance to the field and elegantly provides an update on scientific information on the topic. The figures and tables presented provide relevant additional information.

## AUTHORS' RESPONSE TO THE REVIEWERS

Dear Editor and Reviewers,

We thank you for the opportunity to revise our manuscript, *“Advances and Challenges in the Search for New Treatments for Chagas Disease.”* We are grateful to the reviewers for their careful evaluation and constructive comments. All suggestions have been addressed, and the revised manuscript reflects the requested changes, which we believe have substantially improved its clarity and quality. Our detailed responses to each comment are provided below, and we hope the manuscript is now suitable for publication.

Thank you for your consideration.

Sincerely,

Best regards,

Santi, Alvarenga, and Murta

## REVIEWER COMMENTS: Reviewer: 1

The revised form of the review “Advances and Challenges in the Search for New Treatments for Chagas Disease” has clearly improved. However, few points still need to be addressed:

1. Lines 18-21: Include the references of VNI/VFV studies that are not included and cited

For VNI: Soeiro Mde N, de Souza EM, da Silva CF, Batista Dda G, Batista MM, Pavão BP, Araújo JS, Aiub CA, da Silva PB, Lionel J, Britto C, Kim K, Sulikowski G, Hargrove TY, Waterman MR, Lepesheva GI. *In vitro* and *in vivo* studies of the antiparasitic activity of sterol 14α-demethylase (CYP51) inhibitor VNI against drug-resistant strains of *Trypanosoma cruzi*. Antimicrob Agents Chemother. 2013 Sep;57(9):4151-63. doi: 10.1128/AAC.00070-13.

For VFV: Guedes-da-Silva FH, Batista DDGJ, Da Silva CF, Pavão BP, Batista MM, Moreira OC, Souza LRQ, Britto C, Rachakonda G, Villalta F, Lepesheva GI, Soeiro MNC. Successful Aspects of the Coadministration of Sterol 14α-Demethylase Inhibitor VFV and Benznidazole in Experimental Mouse Models of Chagas Disease Caused by the Drug-Resistant Strain of *Trypanosoma cruzi*. ACS Infect Dis. 2019 Mar 8;5(3):365-371. doi: 10.1021/acsinfecdis.8b00253.

Response: Thank you for the correction, the references were added:

Extensive structural and biochemical evidence has validated TcCYP51 as a druggable enzyme, and several azole-based antifungals, including posaconazole, ravuconazole (and its prodrug fosravuconazole), and VNI/VFV derivatives, have shown potent preclinical activity (84,85).

(…)

Azole Derivatives (e.g., posaconazole, ravuconazole prodrug E1224, VNI): These antifungals demonstrated potent *in vitro* activity. In animal models, posaconazole achieved curing effects in both acute and chronic phases (murine models), and VNI—targeting CYP51—showed complete cure in mice with excellent pharmacokinetic and safety profiles (84).

2. Lines 34-44: Nwoke ET AL., 2024 is a review. Include some references from the cited targets (e.g. cordycepin (3′-deoxyadenosine), 7-(4-chlorophenyl)-3′-deoxy-7 deazadenosine, 9-deazadenosines, and N6-methyltubercidin among others).

Response: Thank you for the correction, we added the references:

This dependency on purine salvage can also be exploited using other nucleoside analogues, including allopurinol ribonucleoside (134), 9-deazainosine (134), formycin B (135), 7-(4-chlorophenyl)-aminopurinol ribonucleoside (136), tubercidin (7-deazaadenosine) (137), cordycepin (3′-deoxyadenosine) (137), tubercidin analogues (138–141), and N6-methyltubercidin (142).

3. Include in item 5.2 (and not in item 6.2 as described in the response letter) the information regarding Brazilian chronic patients who presented high rates of therapeutic failure (not sustained parasitological negativity by qPCR) with the administration of benznidazole in the short-term of drug administration (2 weeks): see at Bosh-Nicolau et al., 2024 LANCET 24:386:394. “…an overall low treatment response in the Brazilian population compared with the other countries. In the control group in the ITT population, the probability of sustained parasitological negativity was 15% (95% CI 5–38) in Brazil compared with 47% (22–73) in Spain, 90% (58–98) in Colombia, and 85% (59–96) in Argentina”.

Response:

We thank the reviewer for this important suggestion. As requested, we have incorporated this information into Section 6.1. We now include the data from Bosch-Nicolau et al. (2024, *The Lancet* 24:386–394) according described below.

6.1 Past Investigations

Azole Derivatives (e.g., posaconazole, ravuconazole prodrug E1224, VNI): These antifungals demonstrated potent *in vitro* activity. In animal models, posaconazole achieved curing effects in both acute and chronic phases (murine models), and VNI—targeting CYP51—showed complete cure in mice with excellent pharmacokinetic and safety profiles (84). However, results were inconsistent across different *T. cruzi* strains, and clinical translation was disappointing (86). E1224 (fosravuconazole, prodrug of ravuconazole) advanced to clinical studies but did not result in successful long-term cure and is no longer pursued. DNDi’s proof-of-concept trial in adults with chronic indeterminate CD showed transient parasite suppression with E1224 monotherapy and sustained responses with benznidazole, supporting the strategy of shorter BZ regimens and selective combinations rather than azole monotherapy (87).

Fexinidazole was elected from a large-scale screening of nitroimidazoles, fexinidazole proceeded to Phase II clinical trials in patients with chronic indeterminate CD. While the drug was well tolerated at lower, short-duration regimens, the efficacy was insufficient to match the standard of care (247). Consequently, DNDi halted its standalone development, although exploration of combination therapies or niche indications (e.g., immunocompromised populations) remains under review.

The BENEFIT trial (Brazil) was study designed to evaluate whether BZ could reduce clinical outcomes in patients with chronic Chagas’ cardiomyopathy. The results demonstrated that while BZ reduced parasitemia in patients with established cardiomyopathy, it did not prevent disease progression over five years (248).

The MULTIBENZ trial evaluated whether alternative benznidazole regimens could achieve efficacy comparable to the standard treatment (300 mg/day for 60 days) for chronic Chagas disease while improving safety (249). The study demonstrated that reducing treatment duration from 8 to 2 weeks could preserve efficacy while enhancing adherence and expanding treatment coverage. However, treatment response was markedly lower in Brazil than in other participating countries. In the control group, sustained parasitological negativity was observed in only 15% of Brazilian patients, compared with 47% in Spain, 90% in Colombia, and 85% in Argentina (249). The authors attributed this disparity to intrinsic parasite-related factors, consistent with previous reports of reduced treatment efficacy in Central Brazil, the region from which the Brazilian patients originated (249).

BZ remains the standard of care and has been an active comparator in most modern trials. DNDi’s BENDITA Phase 2 trial in Bolivia showed that shorter BZ regimens (including 2- and 4-week courses) achieved high rates of sustained qPCR negativity at 6 months with improved tolerability versus the traditional 8-week course; these results informed ongoing work to optimize duration and dosing (250).

6.2 Current and Emerging Efforts

The ongoing NuestroBen trial (Argentina) is a phase III, randomized, multicenter study designed to determine if shorter (2- and 4-week) BZ regimens are equal or better in efficacy than the standard 8-week course for patients with indeterminate or mild cardiac Chagas disease. Another objective is to assess whether these short regimens improve drug tolerability and treatment adherence. The trial is estimated to be completed by September 2026 (251).

4. Revised the references accordingly with the revised MS.

Thank you for pointing this out, the references were revised.

## Reviewer 2

The authors adequately addressed all points raised by the reviewers, making the manuscript more accurate. The manuscript has high scientific relevance to the field and elegantly provides an update on scientific information on the topic. The figures and tables presented provide relevant additional information.

We sincerely thank the reviewer for the positive and thoughtful evaluation of our manuscript. We greatly appreciate the recognition of its scientific relevance, the clarity of the revisions, and the added value of the figures and tables. Your constructive feedback has been invaluable in improving the overall quality and accuracy of the work.

# Reviewer report: REVIEWERS COMMENTS

## REVIEWER #1

No comments.

## REVIEWER #2

No comments.
